# A potential role for rare species in ecosystem dynamics

**DOI:** 10.1038/s41598-019-47541-6

**Published:** 2019-07-31

**Authors:** Torbjörn Säterberg, Tomas Jonsson, Jon Yearsley, Sofia Berg, Bo Ebenman

**Affiliations:** 1 Linköping University, Department of Physics, Chemistry and Biology, Division of Theoretical Biology, SE-58183 Linköping, Sweden; 2 Swedish University of Agricultural Sciences, Department of Aquatic Resources, Skolgatan 6, SE-74242 Öregrund, Sweden; 3 Swedish University of Agricultural Sciences, Department of Ecology, Box 7044, SE-75007 Uppsala, Sweden; 4 Skövde University, Systems Biology Research Centre, Ecological Modelling Group, Box 408, SE-54128 Skövde, Sweden; 5 University College Dublin, School of Biology & Environmental Science & UCD Earth Institute, Belfield, Dublin 4, Ireland; 6 Stockholm University, SRC, SE-10691 Stockholm, Sweden

**Keywords:** Food webs, Ecological modelling, Ecological networks, Theoretical ecology

## Abstract

The ecological importance of common species for many ecosystem processes and functions is unquestionably due to their high abundance. Yet, the importance of rare species is much less understood. Here we take a theoretical approach, exposing dynamical models of ecological networks to small perturbations, to explore the dynamical importance of rare and common species. We find that both species types contribute to the recovery of communities following generic perturbations (i.e. perturbations affecting all species). Yet, when perturbations are selective (i.e. affects only one species), perturbations to rare species have the most pronounced effect on community stability. We show that this is due to the strong indirect effects induced by perturbations to rare species. Because indirect effects typically set in at longer timescales, our results indicate that the importance of rare species may be easily overlooked and thus underrated. Hence, our study provides a potential ecological motive for the management and protection of rare species.

## Introduction

The ecological importance of common species is relatively well documented^1–3^. Yet, the ecological and functional role of rare species, which make up the overwhelming majority of species in the world’s ecosystems^4^, is less well known^5^. There are, however, studies clearly demonstrating the keystone role played by some rare species^6–8^ and their significant contribution to the functional diversity of ecosystems^9–12^. Yet, the relative importance of rare versus common species for the stability of ecosystems in general is largely an open question.

Theoretically, the relative ecological role of rare and common species has received little attention. So far, however, in dynamical models of ecological networks, i.e. networks describing who interacts with whom in an ecosystem, enforced extinctions of common species has been shown to lead to larger community wide effects than extinctions of rare species^13,14^. It has also been found that functional extinctions – where an increased mortality rate and subsequently reduced abundance of a given species first leads to an extinction of another species in the community rather than the species itself – are more likely to occur if the perturbation affects a ‘high biomass’ (i.e. common) species compared to a ‘low biomass’ (i.e. rare) species^15^. Similarly, harvesting a species at maximum sustainable yield has been shown to lead to larger community wide effects if harvesting is applied to common compared to rare species^16^. All these studies indicate that when large and sustained selective perturbations affect common species it will lead to larger community wide effects than if such perturbations affect rare species. However, the methods used in these studies are not directly suitable for evaluating the relative dynamical importance of rare and common species, since these type of perturbations may be considered large from a community point of view when applied to some species, and small when applied to others. For example, selective removal of a high biomass species may be considered a much larger perturbation to a community than removal of a low biomass species. Thus, thoroughly comparing the relative dynamical importance of rare and common species in an unbiased way requires a more sophisticated approach. To this end, an alternative is to investigate how small perturbations, assumed to be of equal magnitude across species, affects the stability properties of a community. Analytical methods for addressing the response to such perturbations have been derived^17–20^, yet the relative effect of perturbing the density of rare and common species has not been thoroughly investigated [but see^20^].

Here we use a theoretical approach, combining analytical and numerical methods, to investigate the relative importance of rare and common species for the stability properties of ecological network models. First, we investigate the relative role of rare and common species in governing the rate of return to equilibrium following generic pulse perturbations; that is, temporary perturbations simultaneously affecting all species in a community. Second, we investigate the recovery of ecological networks exposed to selective pulse perturbations (i.e. temporary perturbations that affect one species at a time) to rare and common species, respectively. Third and finally, we investigate community wide responses of small selective press perturbations, that is, permanent perturbations, to rare and common species. Overall, our analytical and numerical modeling results suggest that rare species may be of particular importance for the stability of ecological communities as communities recover more slowly following pulse perturbations to rare than common species, and because stronger indirect effects are induced if rare rather than common species are permanently perturbed.

## Results

### The relative role of rare and common species in governing the recovery of ecological networks following generic pulse perturbations

Consider the dynamics of a system of *n* interacting species:

1 $$\frac{d{N}_{i}}{dt}={N}_{i}({r}_{i}+{\alpha }_{ii}{N}_{i}+{\sum }_{i\ne j}{\alpha }_{ij}{N}_{j}),$$

where *dN*_*i*_/*dt* is the rate of change in density of species *i*, *N*_*i*_ is the density of species *i*, *r*_*i*_ is the intrinsic growth rate of species *i*, *α*_*ii*_ is the per capita interaction strengths within species and *α*_*ij*_ is the per capita interaction strength between species *j* and *i* (i.e. the effect of species *j* on species *i*).

Following a linearization around an equilibrium point of the non-linear system (Eq. 1) the dynamics of small pulse perturbations (i.e. temporary perturbations) can be studied (Methods). If such perturbations eventually decay, the equilibrium point is called locally stable. Here we investigate the recovery process towards such locally stable equilibria (See Supplementary Fig. 1 for a conceptual illustration of this approach), using both analytical and numerical methods, and investigate the relative dynamical importance of rare and common species during the recovery process.

If it is assumed that a generic pulse perturbation, on average, affects each species in a community to an equal extent, the median initial recovery rate (i.e. just after the perturbation has been imposed [at *t* = 0^+^]) is given by (See Methods & Supplementary Fig. 1):

2 $${R}_{Init}=-\,\frac{1}{n}{\sum }_{i=1}^{n}{\alpha }_{ii}{\hat{N}}_{i},$$

where *R*_*Init*_ is the median recovery rate of the system at time *t* = 0^+^, α_*ii*_ is intra-specific competition and ${\hat{N}}_{i}$ is the equilibrium density of species *i*. If the per capita intra-specific competition among species in a community is assumed to be approximately equal, this equation illustrates that the median initial return rate is primarily governed by common species. Yet, if there is a strong negative correlation between the density of species and their intra-specific competition coefficients this result does not hold.

Furthermore, the initial return rate to equilibrium following a pulse perturbation is only an initial snap-shot (i.e. at time *t* = 0^+^) of the whole recovery process (See Supplementary Fig. 1). Other species may therefore be dynamically important during the rest of the recovery process. As a pulse perturbation decays it will eventually (asymptotically) decay at a rate given by the dominating eigenvalue, *λ*, of the Jacobian matrix evaluated at an equilibrium of the system in question (See Supplementary Fig. 1). This return rate – one measure of a system’s resilience – has frequently been used as a measure of ecological stability^20–22^. For systems close to instability, that is, systems that are particularly vulnerable to perturbations, *λ* will be close to zero and resilience, *Λ* = −Re(*λ*), can be approximated by (See Supplementary Information Section 1 for derivation):

3 $$\Lambda \approx -\,D{({\sum }_{i=1}^{n}\frac{{D}_{-i}}{{\hat{N}}_{i}})}^{-1}$$

Here *D* is the determinant of the Lotka-Volterra interaction matrix **A** with elements *α*_*ij*_ (Eq. 1), and *D*_-*i*_ is the determinant of the reduced matrix **A**_−i_ with the *i*th row and column removed from **A**. Equation (3) shows that resilience is a weighted harmonic mean of species equilibrium densities, and therefore is primarily governed by the densities of rare species. Now, if species 1 is very rare compared to the other species, resilience can be further approximated by $\Lambda \approx -\,D{\hat{N}}_{1}/{D}_{-1}$ (See Supplementary Information Section 1 for details). This shows that resilience is most sensitive to changes in the equilibrium density of the rarest species in a community. Yet, if the dominating eigenvalue is complex, resilience is instead most sensitive to changes in the equilibrium density of the second rarest species in the community (See Supplementary Information Section 1). It is further worth noting that the dominating eigenvalue is not only important for species dynamics at longer timescales. In relation to other eigenvalues it also has the largest overall influence on species densities throughout the recovery process, and its relative importance increases over time (See Supplementary Information Section 2).

**Figure 1:**
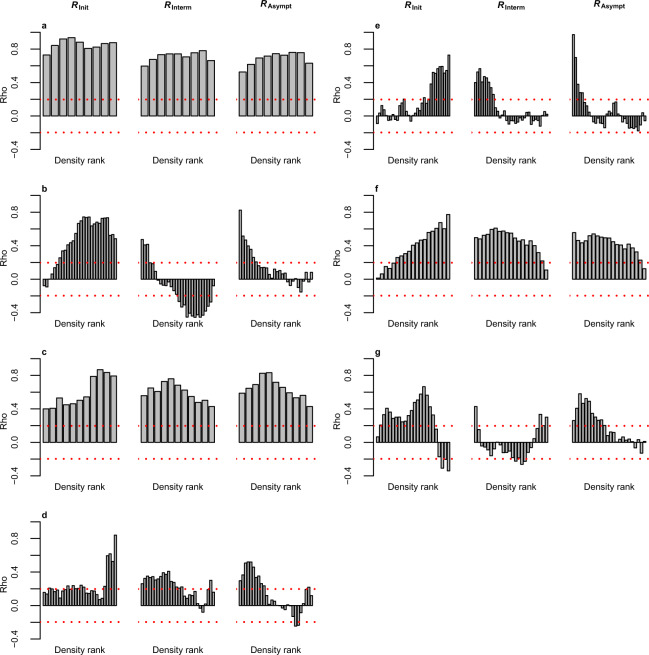
The relative role of common and rare species for the recovery dynamics of model food webs changes over the recovery process. (**a**) Baltic Sea; (**b**) Broadstone stream; (**c**) Lake Vättern; (**d**) Mountane forest; (**e**) Skipwith Pond; (**f**) Tropical Sea and (**g**) Treelease Woods. This figure shows how three different return rates (i.e. initial, intermediate and asymptotic return rates) following generic pulse perturbations (i.e. temporary perturbations affecting all species in a community) are related to species equilibrium biomasses. Bar-plots show the Spearman rank correlation (Rho) for the association between return rates (*R*_*Init*_ – median return rate immediately after the pulse perturbation is being imposed; *R*_*Interm*_ – median return rate for the case when half of the perturbation has recovered; *R*_*Asympt*_ – asymptotic return rate [Resilience], i.e. return rate as *t* - > ∞) and equilibrium biomass of a species with a given biomass rank, across 100 food web replicates. Bars are from rarest (left) to most common (right) species, with rank being based on equilibrium biomass within a food web replicate. Red dashed horizontal lines show the two tailed threshold level of the correlation coefficient at a significance level of 5%. The correlation between the equilibrium biomass of a species, with a given biomass rank within replicates, and return rates are thus significantly positively or negatively correlated if bars cross any of the two threshold levels (i.e. the red horizontal lines), meaning that these species affect return rates. See Supplementary Fig. 1 for a graphical illustration of the different return rates being used.

We numerically investigated our analytical results, relaxing the assumption of closeness to an instability, using models of seven real food webs (with antagonistic trophic links [Methods & Supplementary Information Section 3]) and a broad range of synthetic bipartite networks with a mixture of mutualistic, antagonistic and competitive interactions (Methods & Supplementary Information Section 4). Across replicates of the food webs we found the median initial return rate (i.e. just after a perturbation has been imposed) often to be related to the equilibrium biomass of the most common species (*R*_*init*_ in Fig. 1). However, median initial return rates also depend on the intra-specific competition being experienced by species in a community (Eq. 2). In the bipartite networks, where intra-specific competition coefficients are different for each species in the network (Methods), there is no clear association between species density and median initial return rate (*R*_*Init*_ in Supplementary Fig. 2). In fact, across replicates of these networks the abundance of the most common species is not significantly correlated to median initial return rate (rightmost bar *R*_*Init*_ in Supplementary Fig. 2). Thus, our numerical analyses illustrate that species density in a community cannot be used as a general predictor of initial return rate to equilibrium. Yet, at longer timescales there is a somewhat less ambiguous relationship between species density and return rate across the investigated systems. For recovery rates at intermediate timescales, i.e. where half of the perturbation has recovered (See Supplementary Fig. 1 & Methods), rare species densities are more strongly associated with return rates than common species (*R*_*Interm*_ in Fig. 1 & Supplementary Fig. 2), and at even longer timescales the significance of rare species is even more pronounced (*R*_*Asympt*_ in Fig. 1 & Supplementary Fig. 2).

### Recovery dynamics following selective pulse perturbations

In order to disentangle the relative effect of perturbations to common and rare species, respectively, we conducted selective pulse perturbations. Initially, i.e. just after a selective perturbations has been imposed (at time *t* = 0^+^), the rate of recovery is given by (See Supplementary Information Section 5 for derivation):

4 $${(\frac{d\Vert {\bf{x}}\Vert }{dt})|}_{t={0}^{+}}=-\,{\alpha }_{ii}{\hat{N}}_{i},$$

where α_*ii*_ is the intra-specific competition coefficient for species *i* and ${\hat{N}}_{i}$ its equilibrium density.

Equation (4) shows that communities in general recover more slowly initially, if selective perturbations affect rare rather than common species. If the initial rate of recovery to equilibrium is considered a relevant measure of ecological stability (a system is more stable if it is approaching the equilibrium faster), communities should be more sensitive to perturbations of rare than common species. Yet, again this result is contingent on the intra-specific competition of species in the community. If there is a strong negative correlation between intra-specific coefficients and the equilibrium densities of species this result does not hold.

**Figure 2:**
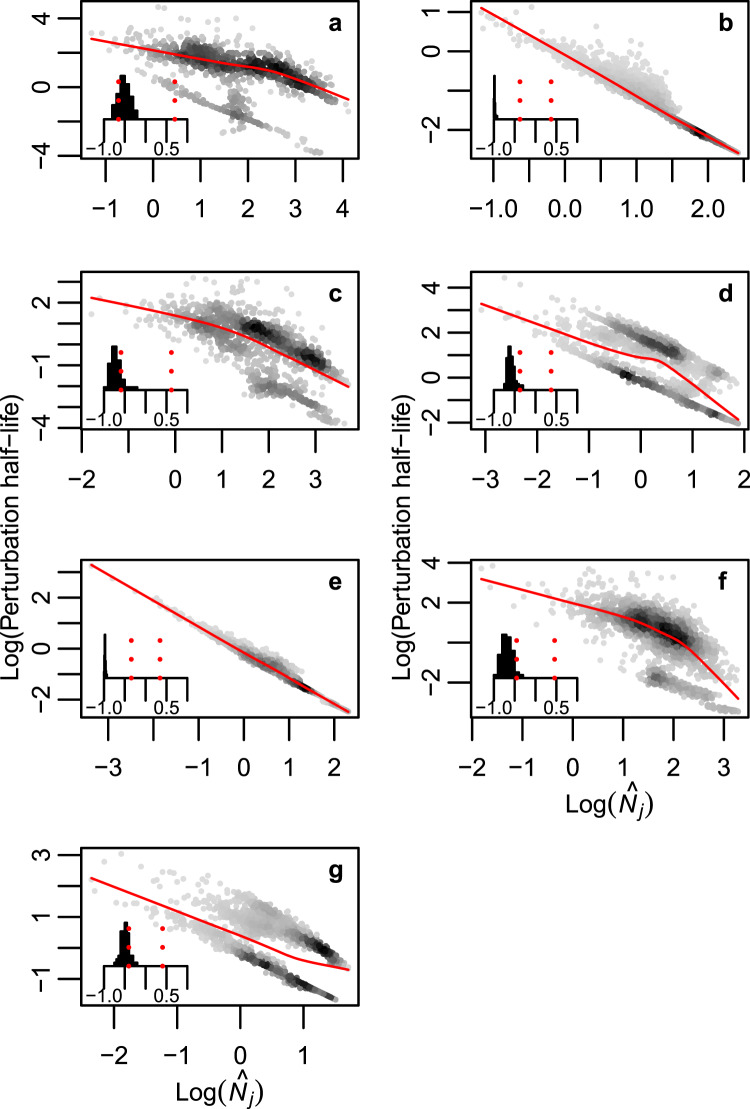
The half-life of selective pulse perturbations are longer if the pulse perturbation affects rare rather than common species in model food webs. (**a**) Baltic Sea; (**b**) Broadstone stream; (**c**) Lake Vättern; (**d**) Mountane forest; (**e**) Skipwith Pond; (**f**) Tropical Sea and (**g**) Treelease Woods. Each data point represents the perturbation half-life as a result of perturbations to one species with equilibrium biomass ${\hat{N}}_{j}$, in one replicate of the food web in question. Density of data points is represented by the grey scale in the scattergrams and the solid red lines are trend lines based on a locally-weighted polynomial regression smoother^48^. Inserted histograms show distribution of Spearman rank correlations between perturbation half-life and species equilibrium biomass across 100 replicates of each food web. Red dashed vertical lines show the two tailed threshold level of the correlation coefficient at a significance level of 5%.

As already mentioned, the initial recovery rate following a pulse perturbation is only an initial snap-shot and may not be representative of the whole recovery process. We therefore numerically investigated the time it takes until a selective perturbation decays to half of its initial magnitude (i.e. a perturbations half-life; See Supplementary Fig. 1 & Methods). Across all systems studied there is a strong and negative association between species equilibrium density and perturbation half-life (Fig. 2 & Supplementary Fig. 3). Indeed, within replicates of food webs as well as bipartite networks there is always a negative relationship between a species density rank and perturbation half-life (small barplots in Fig. 2 & Supplementary Fig. 3).

### Selective press perturbations

**Figure 3:**
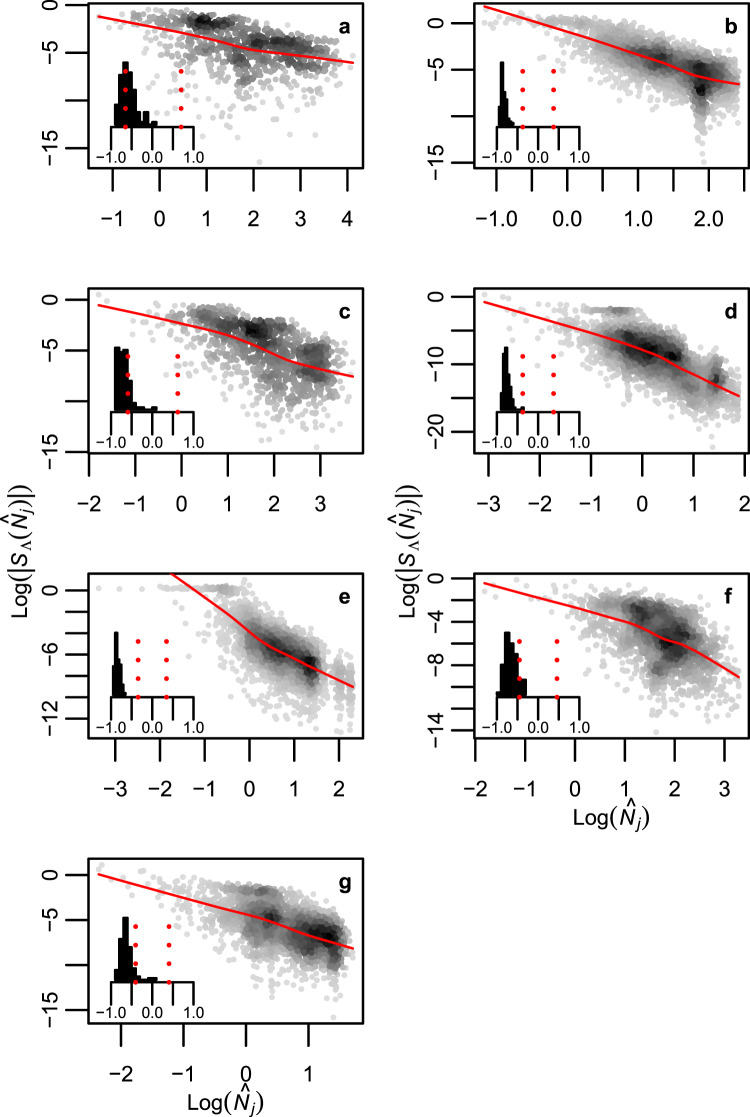
The resilience of model food webs is more sensitive to changes in the equilibrium biomass of rare than of common species. (**a**) Baltic Sea; (**b**) Broadstone stream; (**c**) Lake Vättern; (**d**) Mountane forest; (**e**) Skipwith Pond; (**f**) Tropical Sea and (**g**) Treelease Woods. Density of data points is represented by the grey scale in the scattergrams and the solid red lines are trend line based on a locally-weighted polynomial regression smoother^48^. Each data point represents the sensitivity of resilience (${S}_{\Lambda }({\hat{N}}_{k})$) to a change in the equilibrium biomass of one species (${\hat{N}}_{j}$) in one replicate of the network in question. Inserted histograms show distribution of Spearman rank correlations between sensitivity of network resilience and species equilibrium biomass across 100 replicates of each food web. Red dashed vertical lines show the two tailed threshold level of the correlation coefficient at a significance level of 5%.

**Figure 4:**
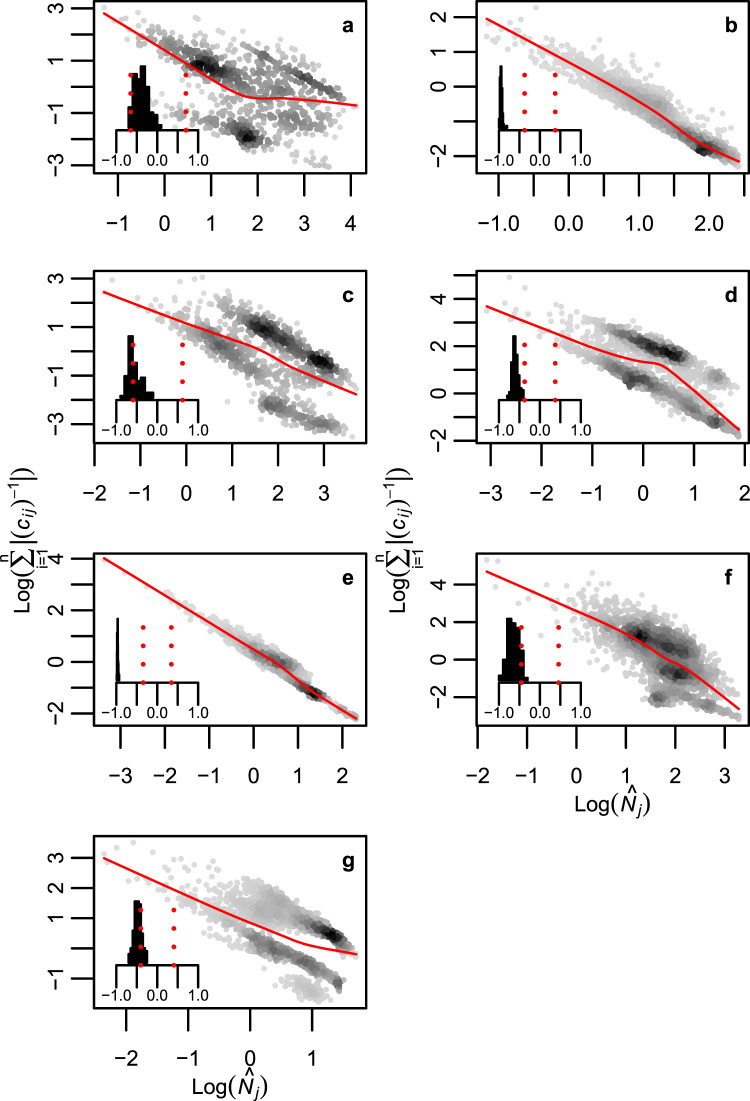
Selective press perturbations lead to larger changes in species equilibrium abundances if the perturbation affects rare rather than common species. (**a**) Baltic Sea; (**b**) Broadstone stream; (**c**) Lake Vättern; (**d**) Mountane forest; (**e**) Skipwith Pond; (**f**) Tropical Sea and (**g**) Treelease Woods. Density of data points is represented by the grey scale in the scattergrams and the solid red lines are trend line based on a locally-weighted polynomial regression smoother^48^. Each data point represents the total effect on all species in a community ($\log ({\sum }_{i=1}^{n}|-{({c}_{ij})}^{-1}|)$) of selective perturbations to one species with equilibrium biomass ${\hat{N}}_{j}$, in one replicate of the network in question. Inserted histograms show distribution of Spearman rank correlations between the total effect of selective perturbations and species equilibrium biomass across 100 replicates of each food web. Red dashed vertical lines show the two tailed threshold level of the correlation coefficient at a significance level of 5%.

To further investigate the relative dynamical importance of rare and common species we conducted small selective press perturbations; that is, small and permanent perturbations (See Methods & Supplementary Fig. 1). These analyses show that asymptotic return rate (i.e. resilience) is more sensitive to changes in the density of rare species, than to changes in the density of common species (Fig. 3 and Supplementary Fig. 4; See also Supplementary Figs 6, 7). Indeed, within network replicates there is a strong and significant negative correlation between the density rank of a species (the rarest species having rank 1) and the effect of a change in its equilibrium density on resilience (small barplots in Fig. 3 and Supplementary Fig. 4; See also Supplementary Figs 6, 7). This result is probably due to the larger changes in species densities that arise from selective press perturbation to rare than common species (Fig. 4 & Supplementary Fig. 5). Indeed, within replicates of the bipartite networks and the seven food webs there is almost always a negative association between species density and the overall direct and indirect effects caused by press perturbation to a given species (small barplots in Fig. 4 & Supplementary Fig. 5). Analytical results further show that this numerical result is general for the class of models studied here, i.e. generalized Lotka-Volterra models (See Supplementary Information Section 6 for derivation).

## Discussion

The dynamical importance of common species, which make up the core of ecological communities, is unquestionably due to their high abundances^1–3^. Yet, the relative dynamical importance of common vs. rare species is less well understood^2^. Theoretically, we here investigated this fundamental ecological question, and found that the importance of rare species may easily be overlooked and thus underrated. Small selective perturbations (assumed to be of equal magnitude across species) to rare species generally led to larger dynamical effects than similar perturbations to common species. This contrasts with previous studies showing that large perturbations, such as species extinctions, led to larger dynamical effects when common species, rather than rare species, were eradicated^13–16^. These apparently conflicting results illustrate that the specific type of perturbation being imposed will affect which specific species that are identified as major contributors to ecological stability^23^. Moreover, the identification of keystones in a community also depends on the specific stability metric being studied^24,25^. Here we used the rate of return to locally stable fixed points as a measure of ecological stability. This definition of stability assumes a static equilibrium point, while empirically based stability metrics are often based on some form of population or community variability^24,25^. Thus, in order to relate our theoretical results to empirical results based on the latter view, it needs to be explored if and how our theoretical stability metrics can be related to population variability. Although this objective warrants future research focus, it is worth noting that there is a direct relationship between the dominating eigenvalue of interaction matrices and population variability in stochastic linear discrete models^26^. This points to the possibility that our results, based on deterministic models, may also hold information for other empirically based stability measures. We have further assumed that species trophic interactions can be described by linear functional responses. The extent to which our results are generalizable to systems with non-linear functional responses therefore remains to be explored. However, it is first worth noting that several theoretical studies have compared model results based on systems with linear and non-linear functional responses, and found that many aspects (such as the probability of observing pyramidal patterns of abundance^27^, the risk of secondary extinctions^15,28,29^, and the probability of local stability of ecological networks^30^) were qualitatively similar for the two scenarios. Secondly, whether linear or non-linear functional forms is a more realistic model of species interactions, in the range of species densities observed in multispecies systems, is an open question^31–33^. Still, to validate if our results holds also under non-linear functional responses, linearization using numerical methods is straight forward^34,35^, and the methods used here could thus easily be adopted to other non-linear systems in future work.

Another important aspect that should be acknowledged is the fact that equilibrium densities are functions of the interaction strengths in a model. One might thus argue that the findings presented here, that is, results illustrating the relative importance of rare vs. common species for the stability of ecological communities, may be confounded by a structured pattern of species interaction strengths (something that may emerge from body size scaling of parameters). Still, although we observe some weak relationships between species interaction strength and equilibrium biomass in the model realizations of the empirical networks (Supplementary Figs 8–10), these relationships are of variable signs for the different networks (positive relationships for some systems and negative for others), indicating that it is not differences in species interaction strengths that are driving the patterns we see in the model food webs. Moreover, for both the bipartite networks, where there are no relationships between equilibrium densities and interaction strengths (Supplementary Fig. 11), and the analytical results, which are to a large extent independent of the specific parametrization used, we found similar results with respect to the relative importance of rare vs. common species. Altogether, we thus think that the results presented here are fairly robust to the specific model parameterization being applied.

Our contribution partly builds on the elegant and informative tools developed by Arnoldi *et al*.^20^ to study short- to long-term recovery of communities following pulse perturbations. Although the major contribution by Arnoldi *et al*.^20^ was to develop tools for studying recovery dynamics of communities following generic pulse perturbations, they also began to explore the relative role of abundant and less abundant species for ecosystem recovery. To this end, they derived an analytical result showing that the rare species in a two species community (where a negative unidirectional effect of a common species on a rare species is assumed) governs asymptotic recovery rate, and found numerically that the rarest species, in a system with competitive interactions only, governs asymptotic recovery rate. In contrast, our study disentangles the relative role of rare and common species for ecological stability at short- to long-time scales, in arbitrarily complex systems (i.e. specious systems with any interaction types), following generic pulse perturbations, selective pulse perturbations as well as selective press perturbations. Using a broad set of analytical as well as numerical methods we have developed the theoretical understanding of the potential relative role of rare and common species for ecological stability by significantly extending the findings of Arnoldi *et al*.^20^, specifically showing that: (i) the long term recovery rate of arbitrarily complex generalized Lotka-Volterra model systems (i.e. with any combination of interaction types) that are close to an instability is governed by rare species and that the contribution of rare species to the recovery process can be significant also at shorter time scales; (ii) press perturbations to rare species lead to an overall larger effect on ecological stability than what perturbations to common species do; (iii) communities are more sensitive to selective pulse perturbations to rare than common species since communities recover more slowly when rare rather than common species are perturbed.

One might argue that our result, showing that communities recover slowly following selective pulse perturbations to rare species, only depends on the fact that rare species approaches equilibrium slowly. By extension, this might be interpreted as if perturbations to rare species only have negligible impact on community dynamics, and only at very long timescales as the rare species recover. Yet, in general we found this not to be true. In fact, (i) rare species have a strong effect on return rates already at intermediate time scales and (ii) stronger indirect effects (i.e. effects on other species mediated via chains of interactions) appear if rare species are perturbed compared to if common species are perturbed (See also Supplementary Information Section 7). Since indirect effects typically set in at intermediate to long timescales^36,37^, our results indicate that the effect of perturbations to rare species may be hidden in ecological dynamics (i.e. not immediately evident and manifested only when indirect effects unfold as time progresses), but the response of rare species perturbations may nevertheless be strong. Therefore, our study indicates that rare species may be just as ecologically important as common species, but the dynamical effects caused by perturbations to rare species may be hard to detect as well as difficult to predict (due to the indeterminacy of ecological interactions involving indirect effects^19^). Thus, our results provides a potential ecological motive for the protection and management of rare species.

## Methods

### Theory on recovery dynamics

The dynamics of a non-linear system, with time derivatives $\frac{d{N}_{i}}{dt}$, close to an equilibrium point, $\hat{{\bf{N}}}$, can be studied using a first order linear approximation:

5 $$\frac{d{\bf{x}}}{dt}={\bf{C}}{\bf{x}},\,\,\,\,{\bf{x}}(0)={{\bf{x}}}_{0}$$

where **C** is the Jacobian matrix of the system evaluated at an equilibrium $(i.e.{c}_{ij}={\frac{{\rm{\partial }}\frac{d{N}_{i}}{dt}}{{\rm{\partial }}{N}_{j}}|}_{\hat{{\bf{N}}}})$, **x** is the displacement from the equilibrium, i.e. ${x}_{i}={N}_{i}-{\hat{N}}_{i}$, and **x**(0) is an initial displacement from the equilibrium at time *t* = 0.

The system of differential equations (Eq. 5) has the particular solution:

6 $${\bf{x}}(t)={e}^{{\bf{C}}t}{{\bf{x}}}_{0},$$

and if the real part of the eigenvalues of **C** are all negative, the initial perturbation eventually decays; that is, *e*^**C***t*^ → 0 *as t* → ∞. Such systems are called locally (or asymptotically) stable. Further, for a locally stable system, a small initial perturbation will eventually (asymptotically) decay at a rate determined by the dominating eigenvalue of **C**, i.e. the eigenvalue with largest real part. This rate of return is sometimes called the resilience of a system:

7 $${\rm{Resilience}}\equiv -\,{\rm{Re}}(\lambda )$$

### Return rates following generic pulse perturbations

In a recent study, Arnoldi *et al*.^20^ developed a set of different return rate metrics that are useful for studying short to intermediate term responses to pulse perturbations of locally stable communities. It is known that resilience is an intrinsic property of a locally stable equilibrium and thus independent of the specific pulse perturbation being imposed. However, at shorter timescales recovery dynamics of any given community depends on the direction of the perturbation. Therefore, Arnoldi *et al*.^20^ developed an approach for investigating the median response across all perturbation directions, for different perturbation scenarios (i.e. assumptions on the relative strength of the initial perturbation on different species). One of these perturbation scenarios assumes Gaussian distributed perturbations that are on average of equal magnitude for all species in a community. We used this specific perturbation model as a null-model to investigate the relative importance of rare and common species for the different parts of the recovery process (from the initial to the asymptotic response) of locally stable ecological models exposed to random pulse perturbations.

#### Median initial return rate (*R*_*Init*_)

Initially, just after a pulse perturbation is being imposed to a stable equilibrium (i.e. at *t* = 0^+^), the median return rate across random perturbation directions *R*_*Init*_ can be described by^20^:

8 $${R}_{Init}=-\,\frac{1}{n}{\rm{tr}}({\bf{C}}),$$

where *n* is the number of species and tr(**C**) is the trace, i.e. the sum of the diagonal elements, of **C**. Now, first assuming Lotka-Volterra dynamics, so that ${c}_{ij}={a}_{ij}{\hat{N}}_{i}$ and ${R}_{Init}=-\,\frac{1}{n}{\rm{tr}}({\bf{C}})=-\,\frac{1}{n}{\sum }_{i=1}^{n}{\alpha }_{ii}{\hat{N}}_{i}$ (where *α*_*ii*_ is Lotka-Volterra intraspecific competition terms and ${\hat{N}}_{i}$ the equilibrium density of species *i*); and secondly assuming that intraspecific competition, *α*_*ii*_, is approximately equal among species in a community; it can be seen that the initial return rate of a community is primarily governed by the abundance of common species. If the latter assumption is not true, other species may be important. Yet, a strong negative correlation between abundance and the strength of intraspecific competition is needed for common species not to have a major impact on the initial response to a perturbation.

#### Median intermediate return rate (*R*_*Interm*_)

Intermediate return rates were found from numerical simulations of food webs and bipartite networks (See below). This was done by recording the time it takes until the magnitude of an initial pulse perturbation recovers to a proportion *c* of its initial size (See Supplementary Fig. 1):

9 $$T(c)=\,\min \,\{t|||{\bf{x}}(t+s)||\le {\rm{c}}\,{\rm{for}}\,{\rm{all}}\,{\rm{s}}\ge 0\},$$

where *T*(*c*) is the time it takes until the magnitude of an initial perturbation has recovered and remains below a certain proportion *c* of its initial size.

For 100 initial random perturbations given by:

10 $${{\bf{x}}}_{0}=\frac{{{\bf{x}}{\boldsymbol{{\prime} }}}_{0}}{\Vert {{\bf{x}}{\boldsymbol{{\prime} }}}_{0}\Vert },$$

where ${{\bf{x}}{\boldsymbol{{\prime} }}}_{{\rm{o}}}$ = $$[\begin{array}{c}{x{\prime} }_{1}\\ \vdots \\ {x{\prime} }_{n}\end{array}]$$ and ${x{\prime} }_{i}$ is a standard normal variable with a mean of zero and standard deviation of unity (i.e. ${x{\prime} }_{i}\sim N(0,1)$), numerical simulations were performed (Eq. 5) and the perturbation half-life (i.e. *T*(*c*) for *c* = 0.5) was recorded for each of these perturbations. The median across these was thereafter used to calculate an average return rate, given by^20^ (See Supplementary Fig. 1):

11 $${R}_{Interm}=-\,\frac{\mathrm{ln}||{\bf{x}}(t)||-\,\mathrm{ln}||{{\bf{x}}}_{0}||}{t},$$

where **x**_0_ is the pulse perturbation and **x**(t) are species’ displacement from equilibrium at time *t*.

#### Asymptotic return rate (*R*_*Asympt*_)

Asymptotic return rate, i.e. resilience, was calculated using the dominating eigenvalue of the Jacobian matrix evaluated at stable equilibria (i.e. using Equation (7)).

#### Numerical analyses of the association between median return rate and species density

Numerical analyses were performed in order to disentangle the relative role of rare and common species for the different parts of the recovery process. First; for each of the 100 replicates of a given system (See below) median initial, intermediate and asymptotic return rates were derived as described above. Second; Spearman rank correlations were calculated for the association, across replicates of a given network, between equilibrium density of a species with a given density rank within a replicate and median return rate.

### Selective pulse perturbations

#### Perturbation half-life

The half-life of a perturbation (Supplementary Fig. 1; Eq. 9) was derived by simulating the recovery dynamics following a selective perturbations of magnitude ||**x**_0_||=1, where the *i*th element of **x**_0_ is equal to one, that is **x**_0_(i) = 1, and all other elements are zero.

### Selective press perturbations

#### Sensitivity of resilience

Sensitivity analyses were applied on both food webs and computer-generated bipartite networks to study the effect of a small change in the equilibrium density of a species (assumed to be caused by changes in the intrinsic growth rates of species) on network resilience. Sensitivity of resilience (*S*_*Λ*_) to a small change in the equilibrium density (${\hat{N}}_{k}$) of species *k* is given by ${S}_{\Lambda }({\hat{N}}_{k})$ = $-\,{\rm{Re}}({\sum }_{j}\frac{{\bar{v}}_{k}{w}_{j}}{{\bf{w}},\,{\bf{v}}}{\alpha }_{kj})$ (See Supplementary Information Section 8 for details). Here *α*_*kj*_ is the per capita effect of species *j* on the per capita growth rate of species *k*, **v** and **w** are the left and right eigenvectors corresponding to the dominant eigenvalue of the Jacobian matrix of the network, *v*_*k*_ and *w*_*j*_ are elements *k* and *j* of the respective vectors, ${\bar{v}}_{k}$ is the complex conjugate of *v*_*k*_, and <**w**, **v**> is the scalar product of **w** and **v**. Elasticity of resilience (*E*_*Λ*_) to a change in the equilibrium density (${\hat{N}}_{k}$) of species *k* is given by ${E}_{\Lambda }({\hat{N}}_{k})$ = $-\,{\rm{Re}}({\sum }_{j}\frac{{\bar{v}}_{k}{w}_{j}}{{\bf{w}},{\bf{v}}}{\alpha }_{kj})(\frac{{\hat{N}}_{k}}{\Lambda })$.

#### The inverse Jacobian matrix

We used the inverse Jacobian matrix, a metric that has been extensively used elsewhere^18,19,34^, to investigate the direct and indirect effects of a small continuous density addition to one species on the equilibrium densities of all species in a community (See Supplementary Information Section 9 for derivation).

We investigated the total effect of a small continuous density addition to species *j* on all species equilibrium densities, given by:

12 $${\sum }_{i=1}^{n}|-{({c}_{ij})}^{-1}|$$

where (*c*_*ij*_)^−1^ is the *i*-*j*th element of the inverse Jacobian matrix **C**^−1^ evaluated at a locally stable equilibrium point.

### Food webs

Seven food webs (See Supplementary Information Section 3 & Supplementary Table 1 for descriptive statistics) were included in the analysis. These food webs were assembled from different ecosystems, and we used information on average body size of trophic groups to parameterize them. Food web networks, i.e. the binary network describing who feeds on whom, and body size information were retrieved from different sources (Food web networks: Baltic Sea^17^, Broadstone Stream^38^, Lake Vättern^17^, Montane Forest^39^, Skipwith Pond^40^, Trelease Woods^39^, Tropic Sea^39^; Body size information: Baltic Sea^17^, Broadstone Stream^41^, Lake Vättern^17^, Montane Forest^42^, Skipwith Pond^41^, Trelease Woods^42^, Tropic Sea^42^). Dynamics of food webs are described by generalised Lotka-Volterra models with linear functional response of consumer species^15,17,30,43^:

13 $$\frac{d{N}_{i}}{dt}={N}_{i}({r}_{i}+{\sum }_{j}{\alpha }_{ij}{N}_{j})$$

Here *N*_*i*_ is the biomass density of species *i* in a community with *n* species, *r*_*i*_ is the intrinsic growth rate of species *i* and ${\alpha {\prime} }_{ij}{\rm{s}}$ are the strengths of interaction between and within species. For systems with linear functional response there is only one internal equilibrium point with all species present. For each natural food web we generated 100 feasible (all species have positive equilibrium densities) and locally stable replicates. Those replicates were generated by varying some of the model parameters (See below and reference^17^). The replicates have a decent variability in equilibrium biomasses (Supplementary Fig. 12) and the difference in equilibrium biomass between the most common and the rarest species in each replicate, across all food webs, is approximately three orders of magnitude ($\log ({{\rm{\max }}}_{j}{\hat{N}}_{j})-\,\log ({{\rm{\min }}}_{j}{\hat{N}}_{j})$ = 2.72 ± 0.61 [Baltic Sea 2.82 ± 0.50; Broadstone Stream 2.45 ± 0.43; Lake Vättern 2.82 ± 0.56; Montane Forest 3.03 ± 0.53; Skipwith Pond 3.12 ± 0.57; Tropic Sea 2.49 ± 0.66; Treelease Woods 2.31 ± 0.51]).

Intrinsic growth rates and strengths of predator-prey interactions were inferred from reported body masses of species using allometric relationships^17,44–47^. Intrinsic growth rates, *r*, of species were related to body mass, *m*, according to ${r}_{i}=\beta {m}_{i}^{-0.25}$ where *β* is a constant being positive for basal species (*β* = 2.1677) and negative for consumer species (*β* = −0.001). For the food webs where information on primary producers were lacking (Broadstone Stream, Montane Forest, Skipwith Pond and Trelease Woods) the lowest trophic levels were modelled as basal resources. Per unit biomass effect of consumer *j* on prey *i* was given by ${\alpha }_{ij}=-\,{k}_{ij}{m}_{j}^{-0.25}{P}_{ij}$, where *k*_*ij*_ is a constant drawn from the uniform distribution [0, 1], *m*_*j*_ is the average body mass of species *j* and *P*_*ij*_ is the preference of species *j* for species *i*^17^. In the Baltic Sea and Lake Vättern the preferences are based on relative gut content data^17^ and for the remaining food webs consumers are assumed to show equal preferences for all their prey species. The per unit biomass effect of prey *i* on consumer *j* is positive and related to the effects of consumers on resources by ${\alpha }_{ji}=-\,e{\alpha }_{ij}$ where *e* is the conversion efficiency drawn from the uniform distribution [0.05, 0.2]. In order to generate feasible models of the natural food webs intraspecific competition (self-limitation) in species above the lowest trophic level, *α*_*ii*_, was set individually for each web (Baltic Sea, *α*_*ii*_ = −0.0005; Broadstone Stream, *α*_*ii*_ = −2.1; Lake Vättern, *α*_*ii*_ = −0.005; Montane Forest, *α*_*ii*_ = −0.01; Skipwith Pond, *α*_*ii*_ = −1.5; Trelease Woods, *α*_*ii*_ = −0.03; Tropic Sea, *α*_*ii*_ = −0.005). For species at the lowest trophic level strength of intraspecific competition was set to −1. Species at the lowest trophic level were also assumed to directly compete with each other and the strength of this interspecific competition, *α*_*ij*_, was weak compared to that of the intraspecific competition and drawn from the uniform distribution [−10^−12^, 0]. Species at higher trophic levels competed with each other indirectly through shared resources.

### Bipartite model networks

Bipartite model networks were generated as in Mougi and Kondoh^30^. The networks consist of a mixture of competitive, mutualistic and antagonistic links, where relative fractions of interaction types are specified before adding dynamics^30^. Network connectance, *C* (defined by *C* = *L/L*_*max*_, where *L* is the number of links and *L*_*max*_ is the maximum number of possible links in a bipartite network) is either low or high (*C* = 0.3 and *C* = 0.7, respectively); the proportion of mutualistic links, i.e. the proportion of the realized links that are mutualistic rather than antagonistic in the networks, is either low (0.3) or high (0.7); and the degree of intraspecific competition among species is either low (*ω* = 0.5), intermediate (*ω* = 1) or high (*ω* = 2). The dynamics is represented by continuous time models with type 1 functional response (See Supplementary Information Section 4 for a detailed model description). These bipartite model networks have a decent variability in equilibrium biomasses (Supplementary Fig. 13) and the difference in equilibrium density between the most common and the rarest species in each replicate, across all networks, is approximately two orders of magnitude ($\log ({\max }_{j}{\hat{N}}_{j})-\,\log ({\min }_{j}{\hat{N}}_{j})\,$= 1.97 ± 0.55 [See Supplementary Table 2 for differences in each networks]).

## Supplementary information

Supplementary Information

## Data Availability

All calculations were performed using Matlab 2018a, and the recovery dynamics was numerically simulated using the stiff ode solver ode23s. Figures were produced using the R software (version 3.4.3). The code generated during the current study are available from the corresponding author on reasonable request.
