# Supplementary material for: A potential role for rare species in ecosystem dynamics
Source: Sci Rep. 2019 Jul 31;9:11107. doi: 10.1038/s41598-019-47541-6 (PMC6668475; doi:10.1038/s41598-019-47541-6)
Supplement: Supplementary file 1 — Supplementary Information [file 41598_2019_47541_MOESM1_ESM.pdf]

SUPPLEMENTARY INFORMATION

A potential role for rare species in ecosystem dynamics

Torbjörn Säterberg, Tomas Jonsson, Jon Yearsley, Sofia Berg & Bo Ebenman*

The supplementary information include:

Supplementary Figures 1-13

Supplementary Tables 1-2

Supplementary Information Sections

1. **The relationship between resilience and species abundances**
2. **Solution to systems of linear differential equations**
3. **Natural food webs**
4. **Bipartite model networks with a mixture of mutualistic and antagonistic interactions**
5. **Initial return rate following selective pulse perturbations**
6. **Analytical result illustrating the relative effect of press perturbations to rare and common species**
7. **Localization of left and right eigenvectors when one species is rare**
8. **Sensitivity of resilience**
9. **Interpretation of the inverse Jacobian matrix**
10. **Supplementary references**

Supplementary Figures

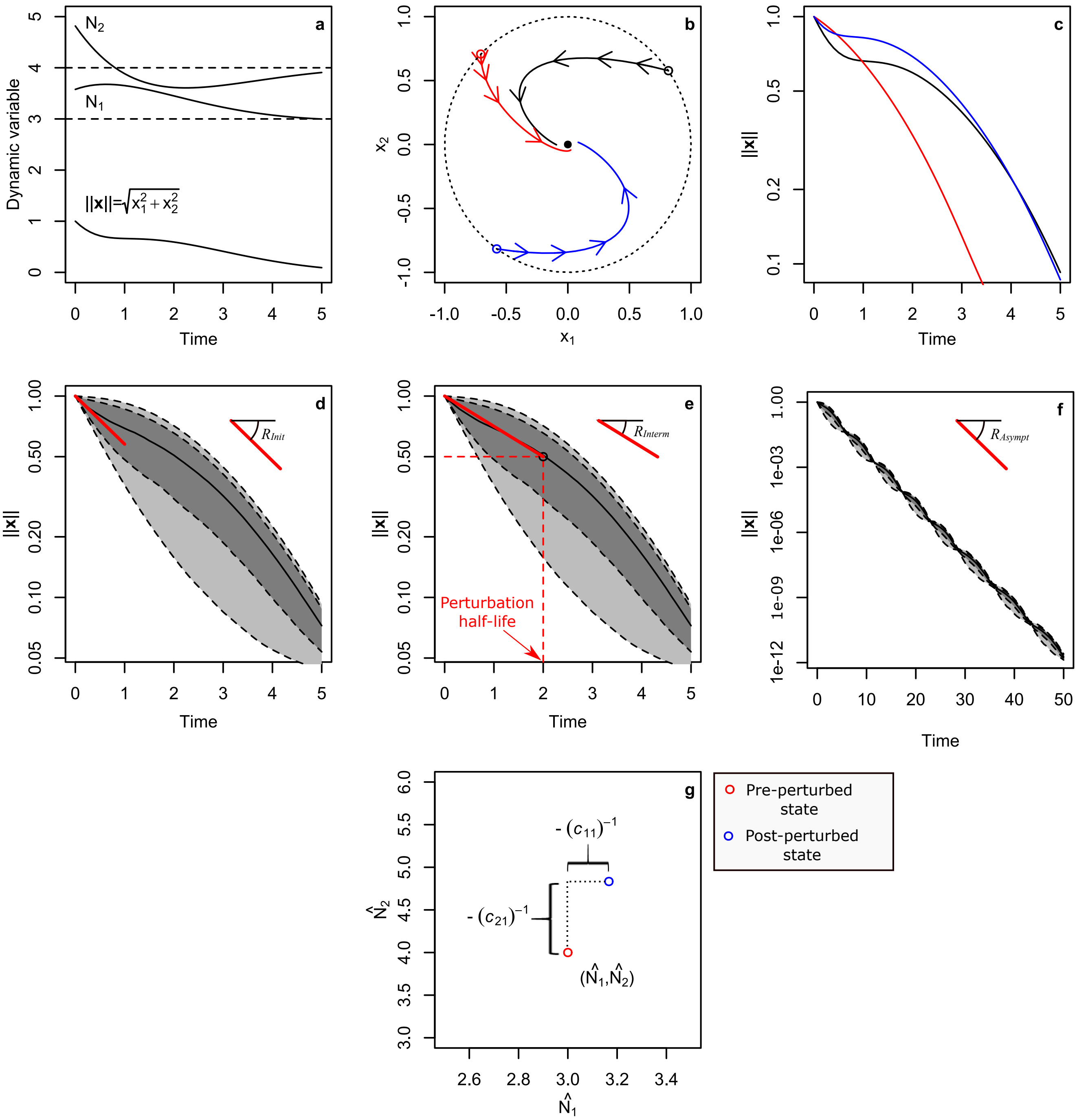

a
N2
N1
||x||=√(x1² + x2²)
Dynamic variable
Time
b
x2
x1
c
||x||
Time
d
RInit
||x||
Time
e
RInterm
Perturbation
half-life
||x||
Time
f
RAsympt
||x||
Time
g
- (c11)−1
- (c21)−1
(N̂1,N̂2)
N̂2
N̂1
Pre-perturbed state
Post-perturbed state

Supplementary Figure 1 | Illustration of the stability metrics used to investigate the relative dynamical importance of rare and common species. Subpanels **a-c** show how a locally stable community (consisting of two species only) recovers following pulse perturbations, i.e. temporary perturbations, of magnitude one (i.e. $||\mathbf{x}_0||=1$). **a**, An example of a time trajectory following a pulse perturbation to a locally stable equilibrium. Dashed lines illustrate equilibrium densities of two species, $\widehat{N}_1 = 3$ and $\widehat{N}_2 = 4$, in a non-linear dynamical system. Time trajectories show densities of the two species and the dynamical variable ($||\mathbf{x}||$) used to investigate recovery dynamics (here $x_i = N_i - \widehat{N}_i$ and $\mathbf{x}_0 = \begin{bmatrix}\sqrt{2/3}\\ \sqrt{1/3}\end{bmatrix}$). **b,** Phase-space plot showing recovery trajectories of a given two-species community exposed to three different pulse perturbations (encoded in blue, red and black, respectively). Dotted circle show initial displacements, i.e. $\mathbf{x}_0$ (all of unit size), arrows indicate the direction of the time trajectories, and the black filled circle in the middle is the equilibrium, that is, $x_1= x_2=0$. **c,** The development over time of the displacement from an equilibrium (as measured by the Euclidean norm) depends on the direction of the initial pulse perturbation (the same colour code and examples as in **b**). **d-g,** Distributions of recovery trajectories following random pulse perturbations assumed, on average, to be of equal magnitude across species (as visualized by the circle in **b**). The solid black line illustrates the median response across these random perturbations, and dashed lines between dark and light grey areas, and between light grey and unshaded areas, correspond to the 50 and 95 percentiles, respectively. **d,** Initial return rates, i.e. the return rate just after a pulse perturbation is being imposed, are given by the tangents of recovery trajectories at time $t=0^+$ (here illustrated by the red thick line) **e,** Intermediate return rates, i.e. the average return rate between time $t=0$ and the time at which half of the initial perturbation has recovered (Perturbation half-life). **f,** Asymptotic return rate (i.e. resilience), the return rate of a community as time goes to infinity ($t \to \infty$). **g,** A graphical illustration of the method used to study press perturbations; that is, permanent perturbations. The

elements of the negative inverse Jacobian matrix encapsulates how a continuous density change (here addition) to one species affects the equilibrium density of all species in a community. The continuous density addition is here assumed to affect species one, leading to a new equilibrium density (blue) of both species in the community. For all subplots we have assumed a Jacobian matrix $\mathbf{C} = \begin{bmatrix} -1 & -1 \\ 0.5 & -0.1 \end{bmatrix}$. This figure was partly adapted from Arnoldi et al.[20].

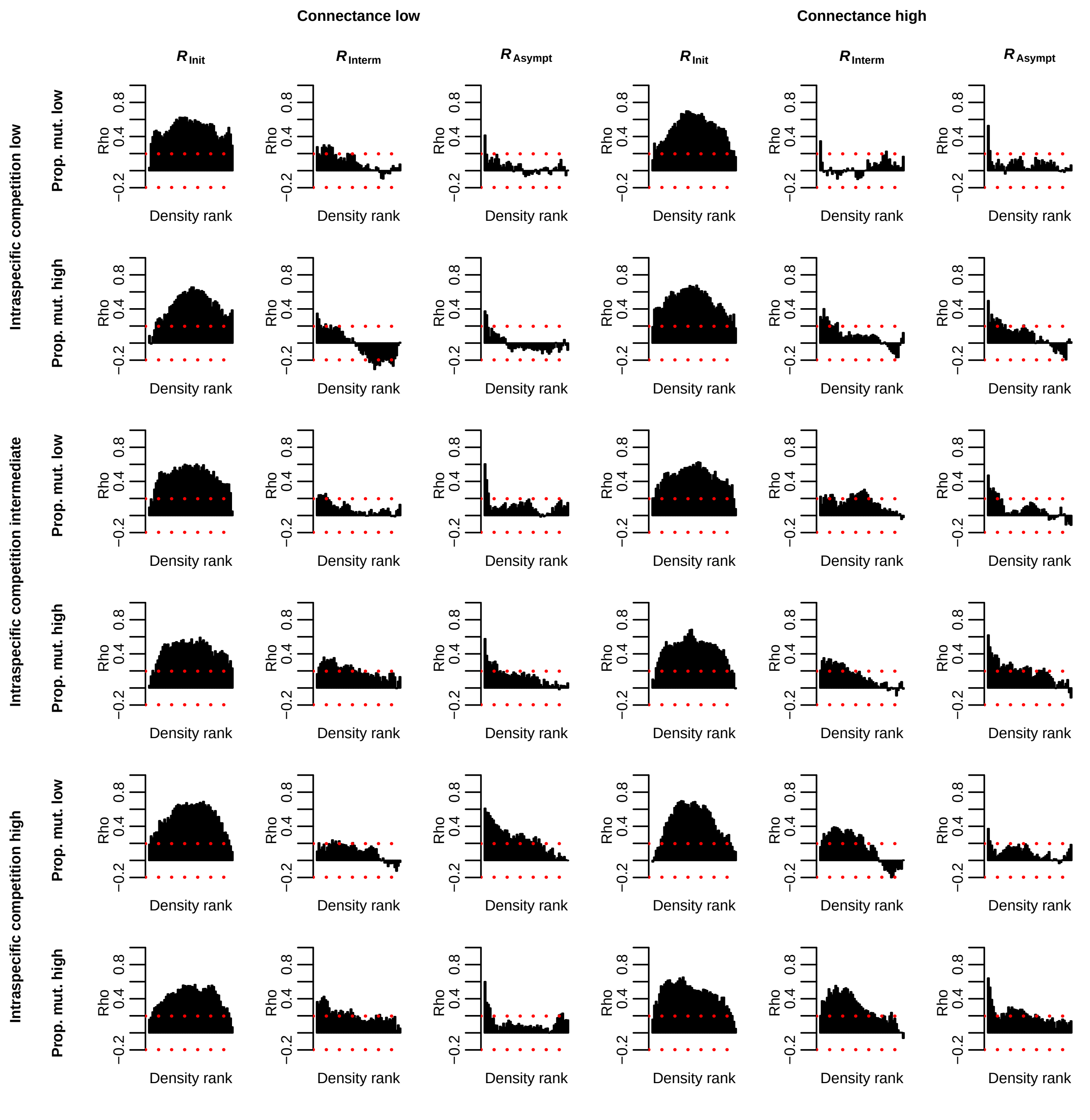

Connectance low
Connectance high
R_{Init}
R_{Interm}
R_{Asympt}
Intraspecific competition low
Intraspecific competition intermediate
Intraspecific competition high
Prop. mut. low
Prop. mut. high
Rho
−0.2
0.4
0.8
Density rank

Supplementary Figure 2 | Rare species govern resilience in bipartite networks. Bar-plots show the Spearman rank correlation (Rho) for the association between return rates (R_{Init} – median return rate just after the pulse perturbation imposed; R_{Interm} – median return rate when half of the perturbation has recovered; R_{Asympt} – asymptotic return rate [Resilience], i.e. return rate as t -> ∞) and equilibrium density of a species with a given density rank, across 100 network replicates. Bars are for rarest (left) to most common (right) species, with rank being based on density within a food web replicate. Red dashed horizontal lines show the two tailed threshold level of the correlation coefficient at a significance level of 5%. Three parameters are varied among the subplots: Connectance – the proportion of possible links that are realised (low=0.3 and high=0.7); proportion of mutualistic links – the proportion of all links where interacting species have positive effects on each other (low=0.3 and high=0.7); intraspecific competition – a scaling parameter, ω, determining the average strength of species self-limitation (ω_{low}=0.5, $\omega_{\mathrm{intermediate}}$=1 and ω_{high}=2).

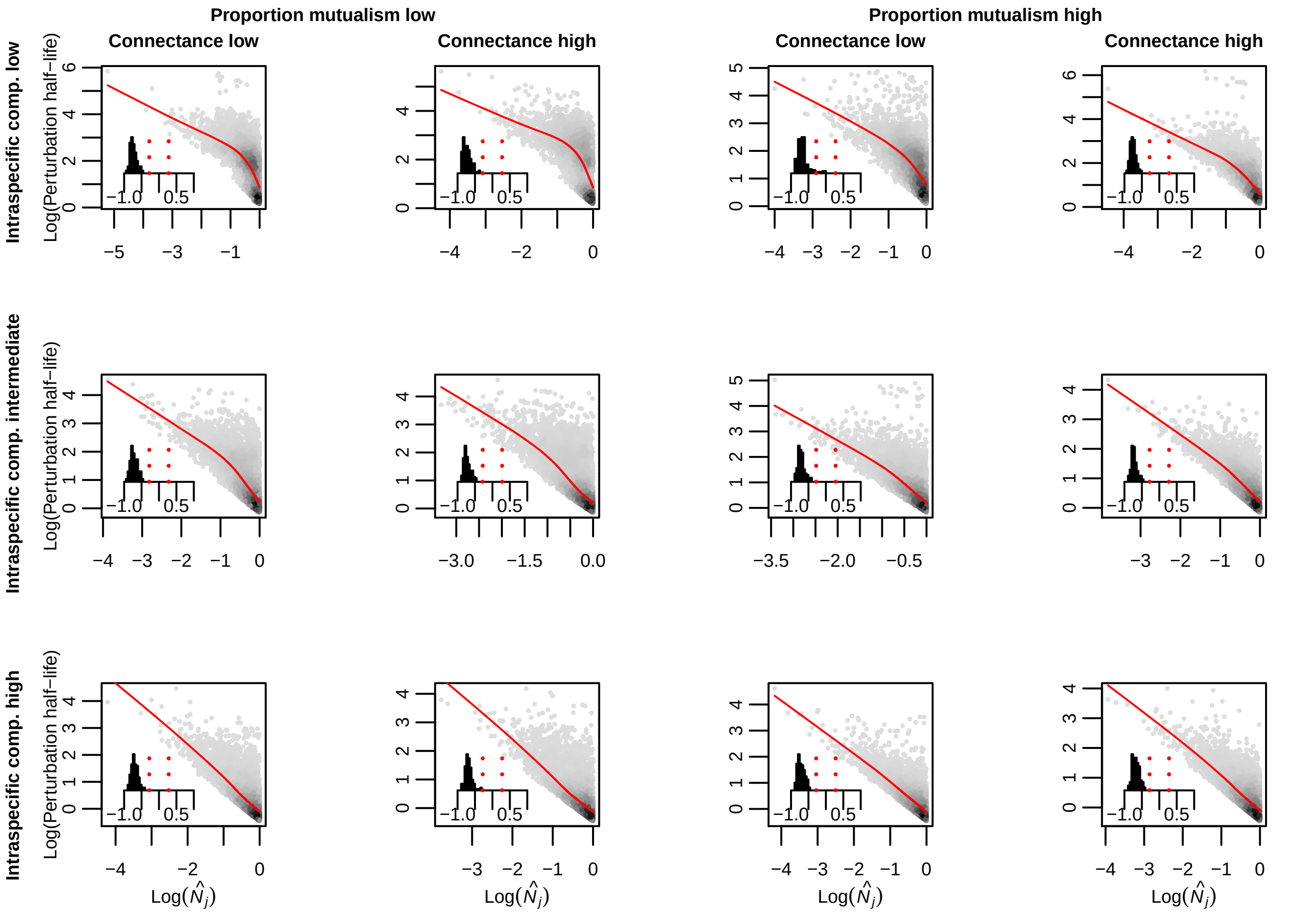

Proportion mutualism low
Proportion mutualism high
Connectance low
Connectance high
Connectance low
Connectance high
Intraspecific comp. low
Intraspecific comp. intermediate
Intraspecific comp. high
Log(Perturbation half−life)
Log($\hat{N}_j$)
−1.0
0.5

Supplementary Figure 3 | The half-life of selective pulse perturbations are longer if the perturbation affects rare rather than common species in bipartite networks. Three parameters are varied among subplots: Connectance – the proportion of possible links that are realised (low=0.3 and high=0.7); Proportion of mutualistic links – the proportion of all links where interacting species have positive effects on each other (low=0.3 and high=0.7); Intraspecific competition – a scaling parameter, ω, determining the average strength of species self-limitation (ω_{low}=0.5, $\omega_{intermediate}$=1 and ω_{high}=2). Density of data points is represented by grey scale and solid red lines are trend lines based on a locally-weighted polynomial regression smoother[43]. Inserted histograms show distribution of Spearman rank correlations between perturbation half-time and species equilibrium density ($\widehat{N}_k$) across 100 replicates of each network. Red dashed vertical lines show the two tailed threshold level of the correlation coefficient at a significance level of 5%.

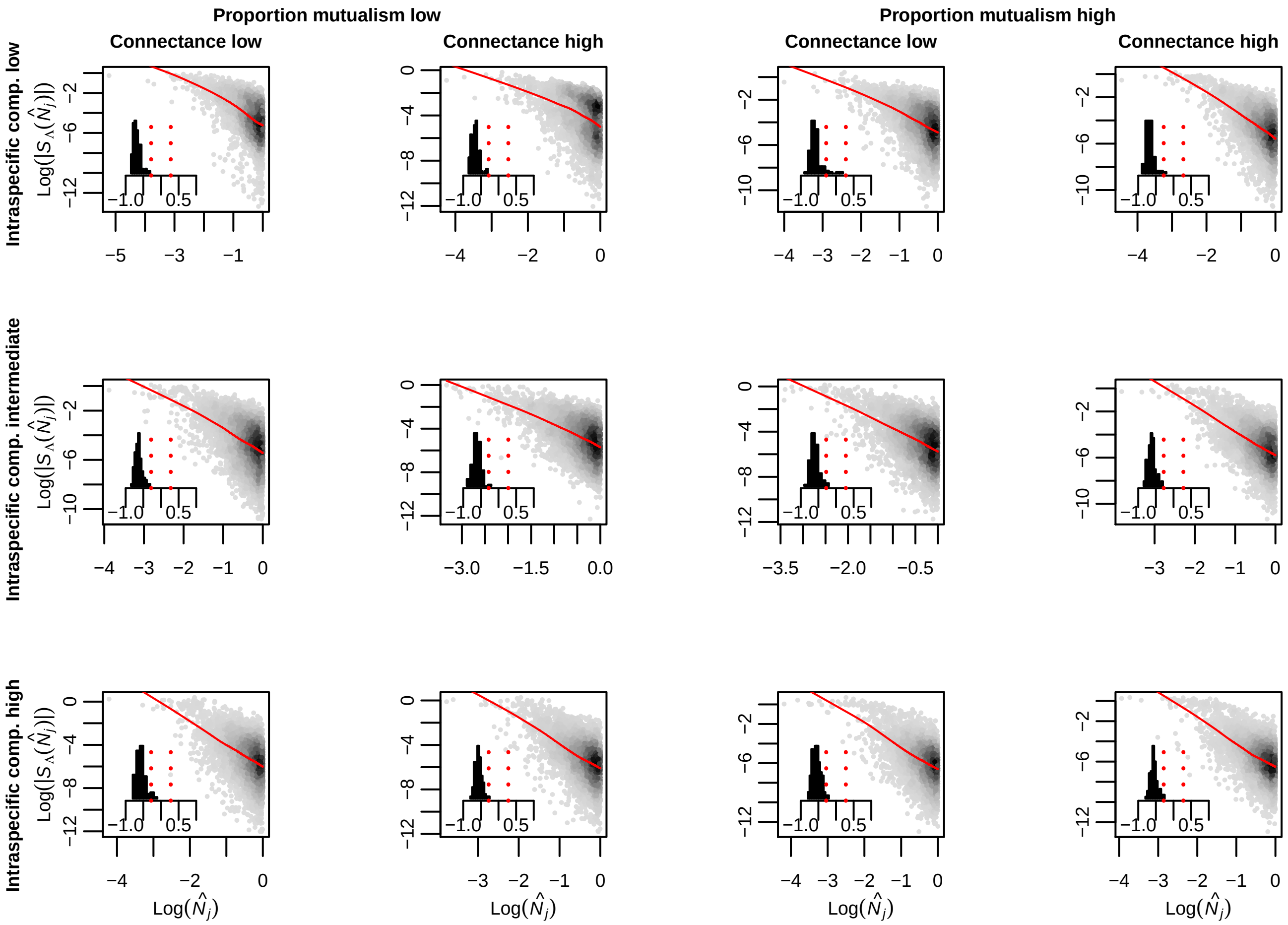

Proportion mutualism low
Proportion mutualism high
Connectance low
Connectance high
Connectance low
Connectance high
Intraspecific comp. low
Intraspecific comp. intermediate
Intraspecific comp. high
Log(|S_Λ(N̂_j)|)
Log(N̂_j)
−1.0
0.5

Supplementary Figure 4 | Resilience is more sensitive to small absolute changes in the abundance of rare than of common species in bipartite model networks. Three parameters are varied among subplots: Connectance – the proportion of possible links that are realised (low=0.3 and high=0.7); Proportion of mutualistic links – the proportion of all links where interacting species have positive effects on each other (low=0.3 and high=0.7); Intraspecific competition – a scaling parameter, ω, determining the average strength of species self-limitation (ω_{low}=0.5, $\omega_{\text{intermediate}}$=1 and ω_{high}=2). Density of data points is represented by grey scale and solid red lines are trend lines based on a locally-weighted polynomial regression smoother[43]. Inserted histograms show distribution of spearman rank correlations between sensitivity of network resilience ($S_\Lambda(\hat{N}_k)$) and species equilibrium density ($\hat{N}_k$) across 100 replicates of each network. Red dashed vertical lines show the two tailed threshold level of the correlation coefficient at a significance level of 5%.

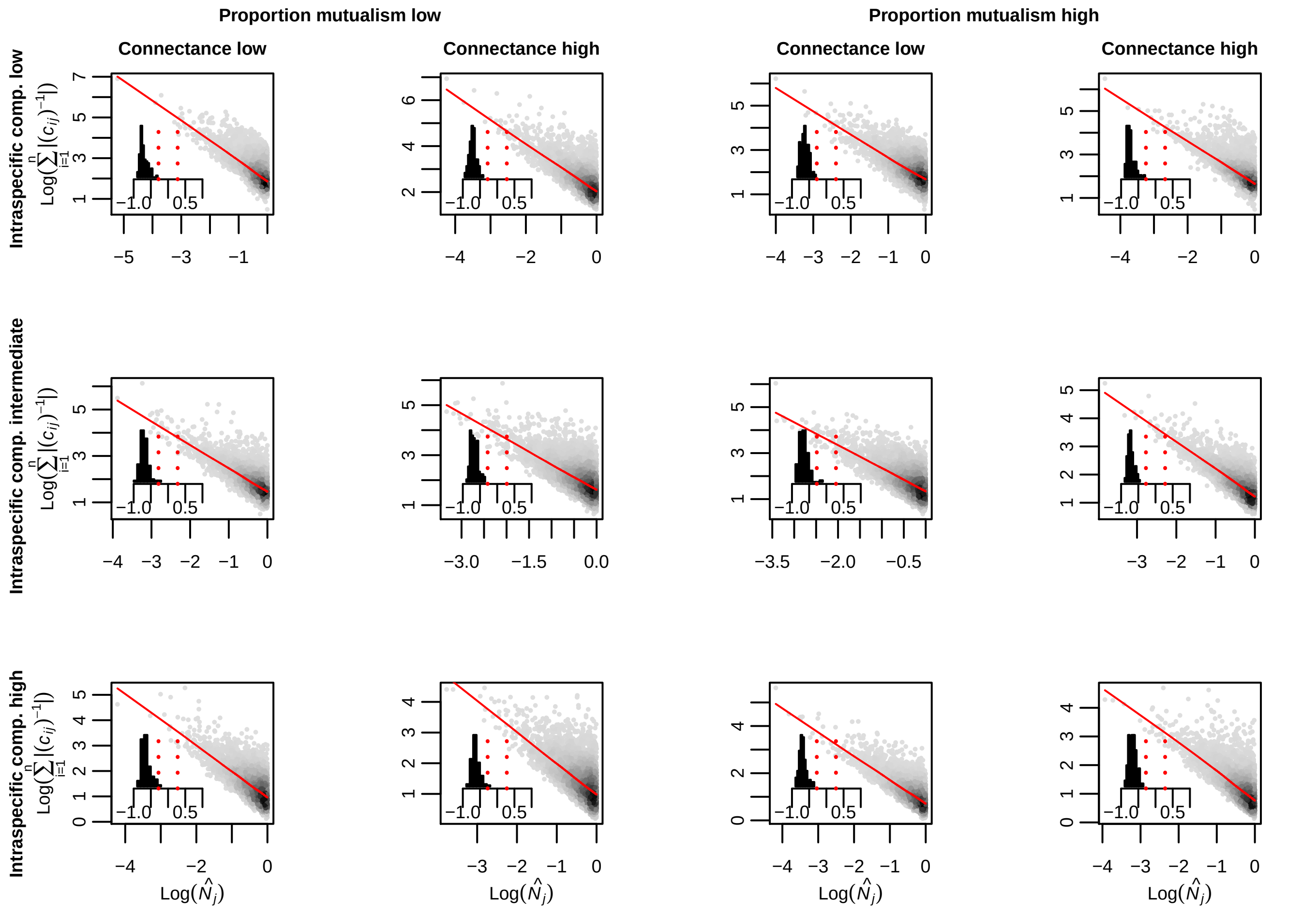

Proportion mutualism low
Proportion mutualism high
Connectance low
Connectance high
Connectance low
Connectance high
Intraspecific comp. low
Intraspecific comp. intermediate
Intraspecific comp. high
$\mathrm{Log}(\sum_{i=1}^{n}|(c_{ij})^{-1}|)$
$\mathrm{Log}(\hat{N}_j)$

Supplementary Figure 5 | Selective press perturbations lead to larger changes in on other species densities if the perturbation is affecting rare rather than common species. Three parameters are varied among subplots: Connectance – the proportion of possible links that are realised (low=0.3 and high=0.7); Proportion of mutualistic links – the proportion of all links where interacting species have positive effects on each other (low=0.3 and high=0.7); Intraspecific competition – a scaling parameter, ω, determining the average strength of species self-limitation (ω_{low}=0.5, $\omega_{intermediate}$=1 and ω_{high}=2). Density of data points is represented by grey scale and solid red lines are trend lines based on a locally-weighted polynomial regression smoother[43]. Inserted histograms show distribution of spearman rank correlations between the total effect of press perturbations ($\log(\sum_{i=1}^{n}\left|-(c_{ij})^{-1}\right|)$) and species equilibrium density ($\widehat{N}_k$) across 100 replicates of each network. Red dashed vertical lines show the two tailed threshold level of the correlation coefficient at a significance level of 5%.

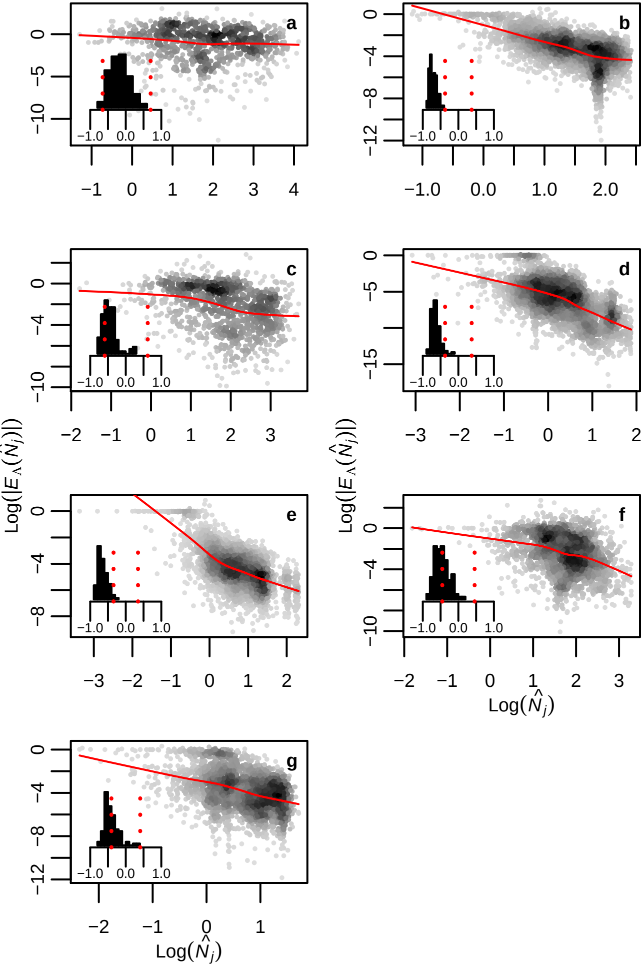
a
b
c
d
e
f
g
Log(|E_Λ(N̂_j)|)
Log(N̂_j)
−1.0
0.0
1.0

Supplementary Figure 6 | The resilience of model food webs is more sensitive to small proportional changes in the abundance of rare than of common species. a, Baltic Sea; **b,** Broadstone stream; **c,** Lake Vättern; **d,** Mountane forest; **e,** Skipwith Pond; **f,** Tropical Sea and **g,** Treelease Woods. Density of data points is represented by the grey scale in the scattergrams and solid red lines are trend line based on a locally-weighted polynomial regression smoother[43]. Each data point represents the elasticity of network resilience ($E_{\Lambda}\left(\widehat{N}_k\right)$) to a change in the equilibrium biomass ($\widehat{N}_k$) of one species in one replicate of the network in question. Inserted histograms show distribution of Spearman rank correlations between elasticity of network resilience and species equilibrium biomass across 100 replicates of each food web. Red dashed vertical lines show the two tailed threshold level of the correlation coefficient at a significance level of 5%.

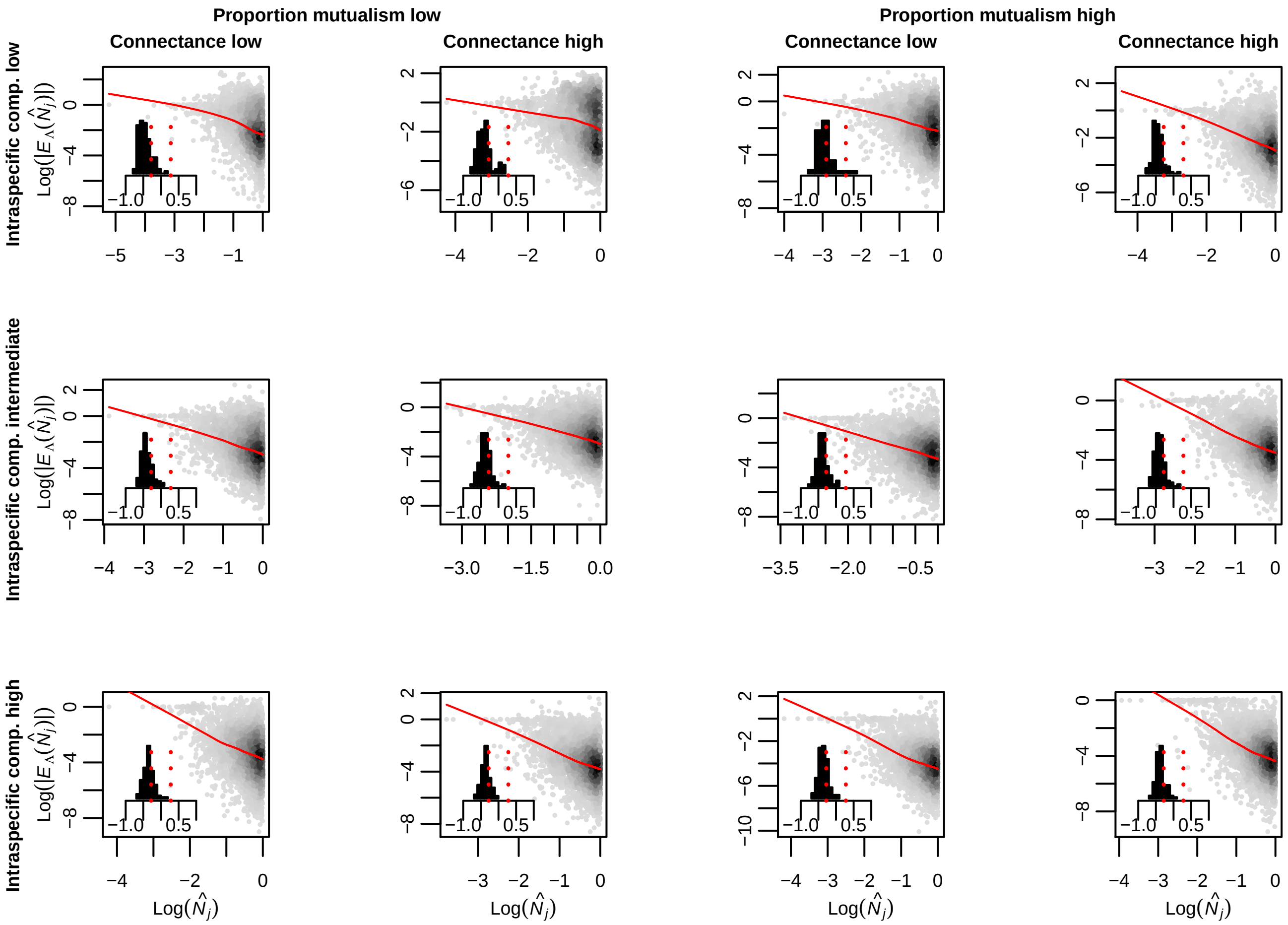

Proportion mutualism low
Proportion mutualism high
Connectance low
Connectance high
Connectance low
Connectance high
Intraspecific comp. low
Intraspecific comp. intermediate
Intraspecific comp. high
$\mathrm{Log}(|E_{\Lambda}(\hat{N}_j)|)$
$\mathrm{Log}(\hat{N}_j)$

Supplementary Figure 7 | Resilience is more sensitive to small proportional changes in the abundance of rare than of common species in bipartite model networks. Three parameters are varied among subplots: Connectance – the proportion of possible links that are realised (low=0.3 and high=0.7); Proportion of mutualistic links – the proportion of all links where interacting species have positive effects on each other (low=0.3 and high=0.7); Intraspecific competition – a scaling parameter, ω, determining the average strength of species self-limitation (ω_{low}=0.5, $\omega_{intermediate}$=1 and ω_{high}=2). Density of data points is represented by grey scale and red solid lines are trend lines based on a locally-weighted polynomial regression smoother[46]. Inserted histograms show distribution of spearman rank correlations between elasticity of network resilience ($E_{\Lambda}(\widehat{N}_k)$) and species equilibrium density ($\widehat{N}_k$) across 100 replicates of each network. Red dashed vertical lines show the two tailed threshold level of the correlation coefficient at a significance level of 5%.

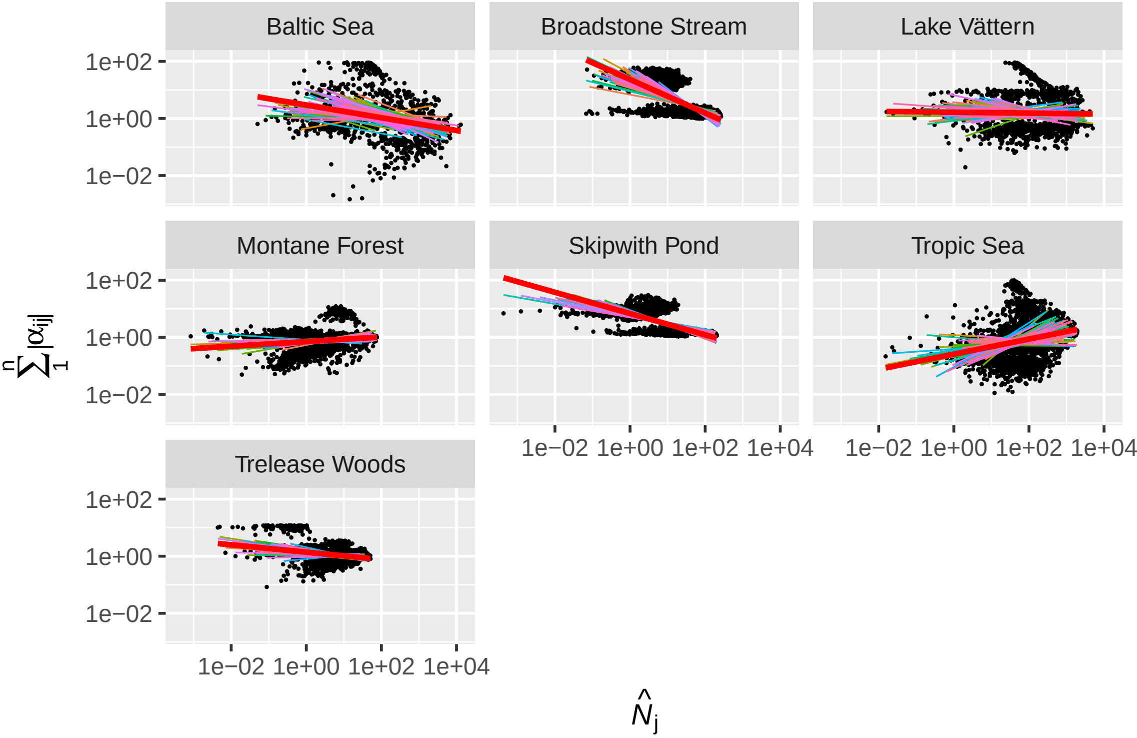
Baltic Sea
Broadstone Stream
Lake Vättern
Montane Forest
Skipwith Pond
Tropic Sea
Trelease Woods
1e+02
1e+00
1e−02
1e−02 1e+00 1e+02 1e+04
$\sum_1^n |\alpha_{ij}|$
$\hat{N}_j$

Supplementary Figure 8 | The relationships between species interaction strength and species equilibrium biomass are different across model food webs. This figure shows the relationship between species equilibrium biomass ($\widehat{N}_j$) and the summed direct effects ($\sum_{i=1}^{n} \alpha_{ij}$) that a given species j has on other species in a community. Black dots represent observations, colored thin lines represent linear trend lines for each food web replicate, and the red thick lines represent linear trend lines across all 100 replicates of each food web.

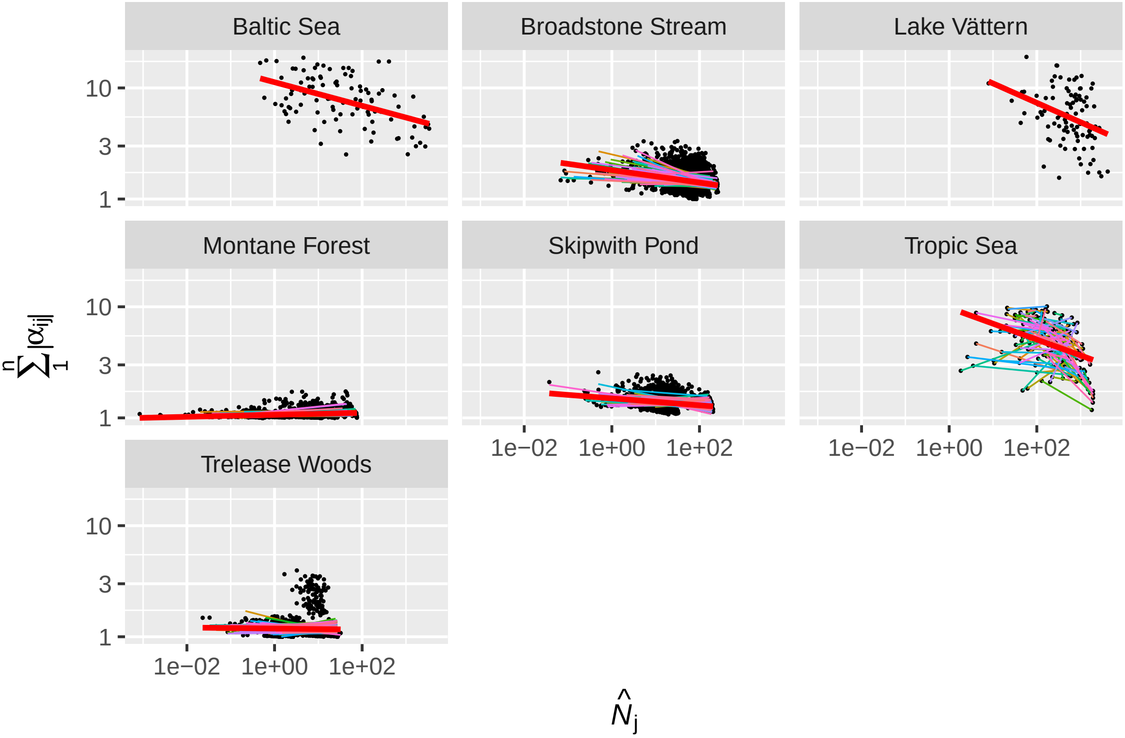

Baltic Sea
Broadstone Stream
Lake Vättern
Montane Forest
Skipwith Pond
Tropic Sea
Trelease Woods
10
3
1
1e−02
1e+00
1e+02
$\sum_{1}^{n} |\alpha_{ij}|$
$\hat{N}_j$

Supplementary Figure 9 | Relationships between species interaction strength and species equilibrium biomass for resource species in model food webs. This figure shows the relationship between species equilibrium biomass ($\widehat{N}_j$) and the summed direct effects ($\sum_{i=1}^{n} \alpha_{ij}$) that a given resource species j (that is, a species which is not feeding on other species in the model) has on other species in a community. Black dots represent observations, colored thin lines represent linear trend lines for each food web replicate, and the red thick lines represent linear trend lines across all 100 replicates of each food web.

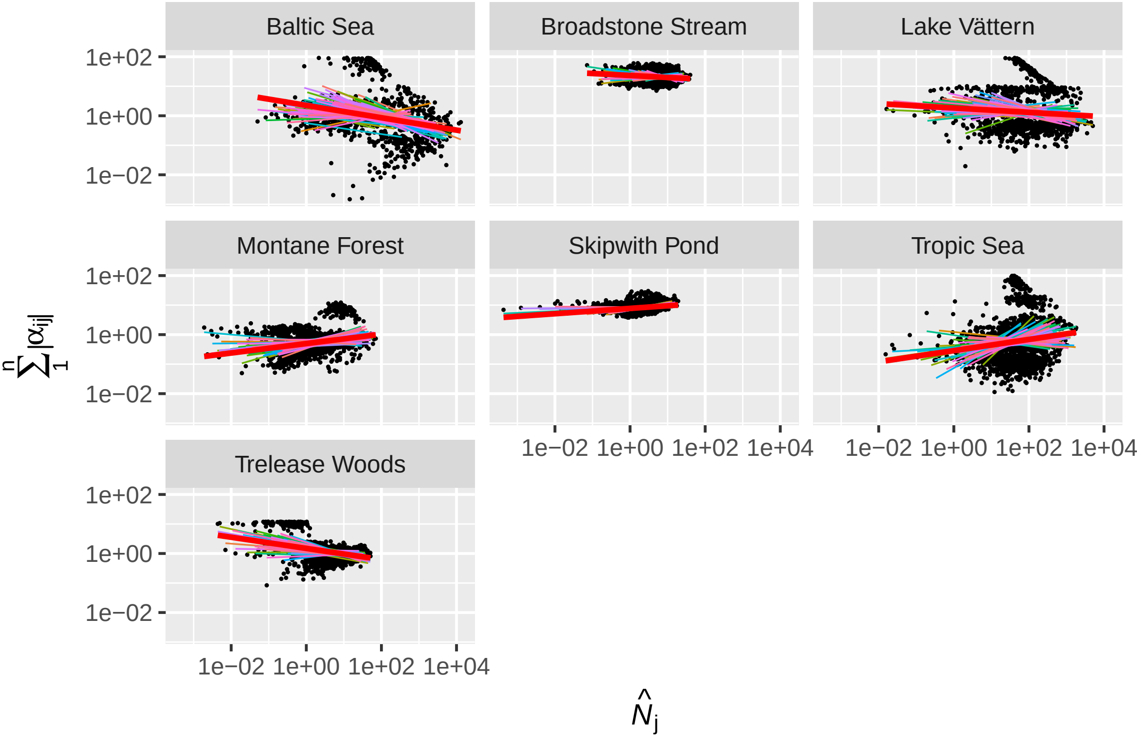

Baltic Sea
Broadstone Stream
Lake Vättern
Montane Forest
Skipwith Pond
Tropic Sea
Trelease Woods
1e+02
1e+00
1e−02
1e−02 1e+00 1e+02 1e+04
$\sum_1^n |\alpha_{ij}|$
$\hat{N}_j$

Supplementary Figure 10 | Relationships between species interaction strength and species equilibrium biomass for consumer species in model food webs. This figure shows the relationship between species equilibrium biomass ($\widehat{N}_j$) and the summed direct effects ($\sum_{i=1}^{n} \alpha_{ij}$) that a given consumer species j (that is, a species which is feeding on other species in the model) has on other species in a community. Black dots represent observations, colored thin lines represent linear trend lines for each food web replicate, and the red thick lines represent linear trend lines across all 100 replicates of each food web.

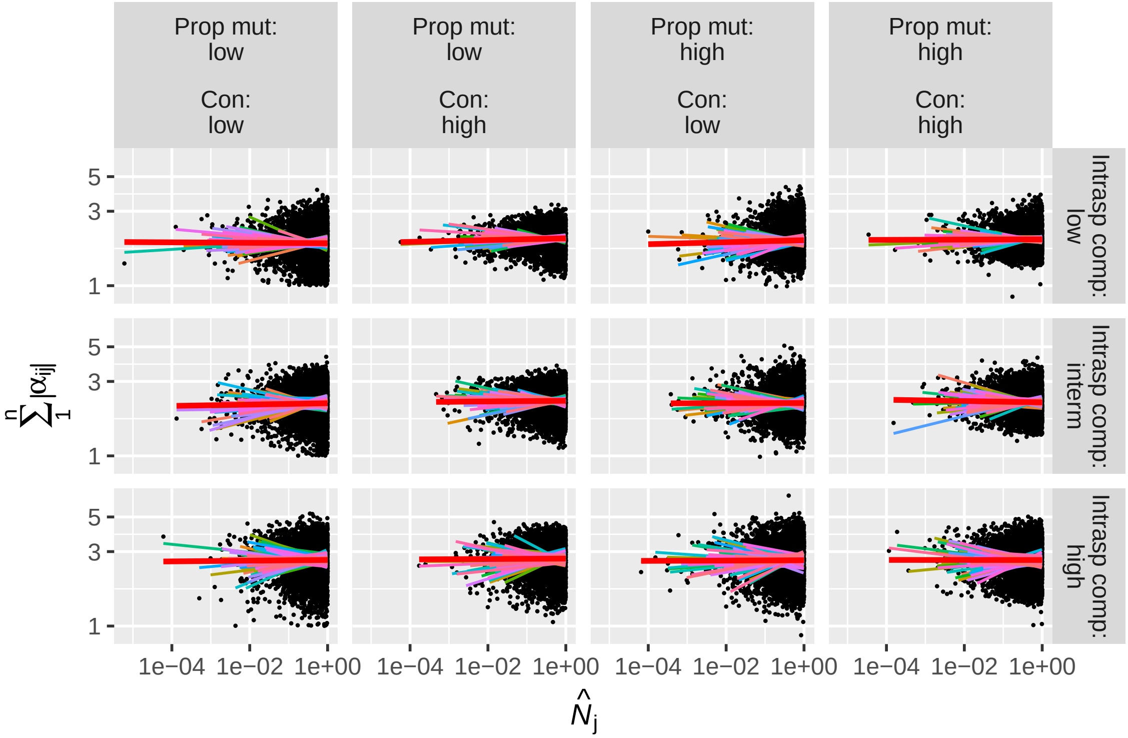

Prop mut: low
Con: low
Prop mut: low
Con: high
Prop mut: high
Con: low
Prop mut: high
Con: high
Intrasp comp: low
Intrasp comp: interm
Intrasp comp: high
5
3
1
1e−04
1e−02
1e+00
$\sum_{1}^{n}|\alpha_{ij}|$
$\hat{N}_j$

Supplementary Figure 11 | Relationships between species interaction strength and species equilibrium density in bipartite networks. This figure shows the relationship between species equilibrium density ($\widehat{N}_j$) and the summed direct effects ($\sum_{i=1}^{n} \alpha_{ij}$) that a given species *j* has on other species in a community. Black dots represent observations, colored thin lines represent linear trend lines for each food web replicate, and the red thick lines represent linear trend lines across all replicates of a given food web. Three parameters are varied among subplots: Connectance – the proportion of possible links that are realised (low=0.3 and high=0.7); Proportion of mutualistic links – the proportion of all links where interacting species have positive effects on each other (low=0.3 and high=0.7); Intraspecific competition – a scaling parameter, ω, determining the average strength of species self-limitation (ω_{low}=0.5, $\omega_{intermediate}$=1 and ω_{high}=2).

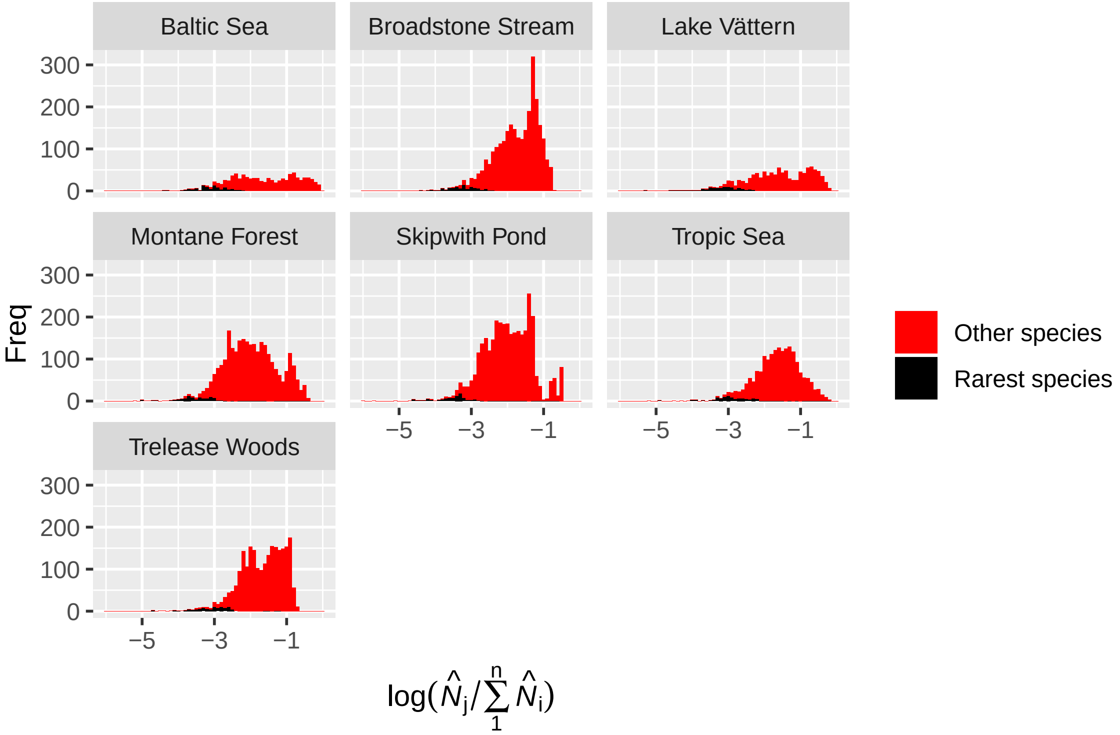

Baltic Sea
Broadstone Stream
Lake Vättern
Montane Forest
Skipwith Pond
Tropic Sea
Trelease Woods
300
200
100
0
Freq
−5
−3
−1
$\log(\hat{N}_j / \sum_1^n \hat{N}_i)$
Other species
Rarest species

Supplementary Figure 12 | Equilibrium biomass distribution in model food webs. This histogram shows the relative distribution of equilibrium biomasses within food web replicates ($\log\left(\frac{\hat{N}_j}{\sum_i \hat{N}_i}\right)$), for each model food web. The rarest species in each food web replicate is coded in black and other species are coded in red.

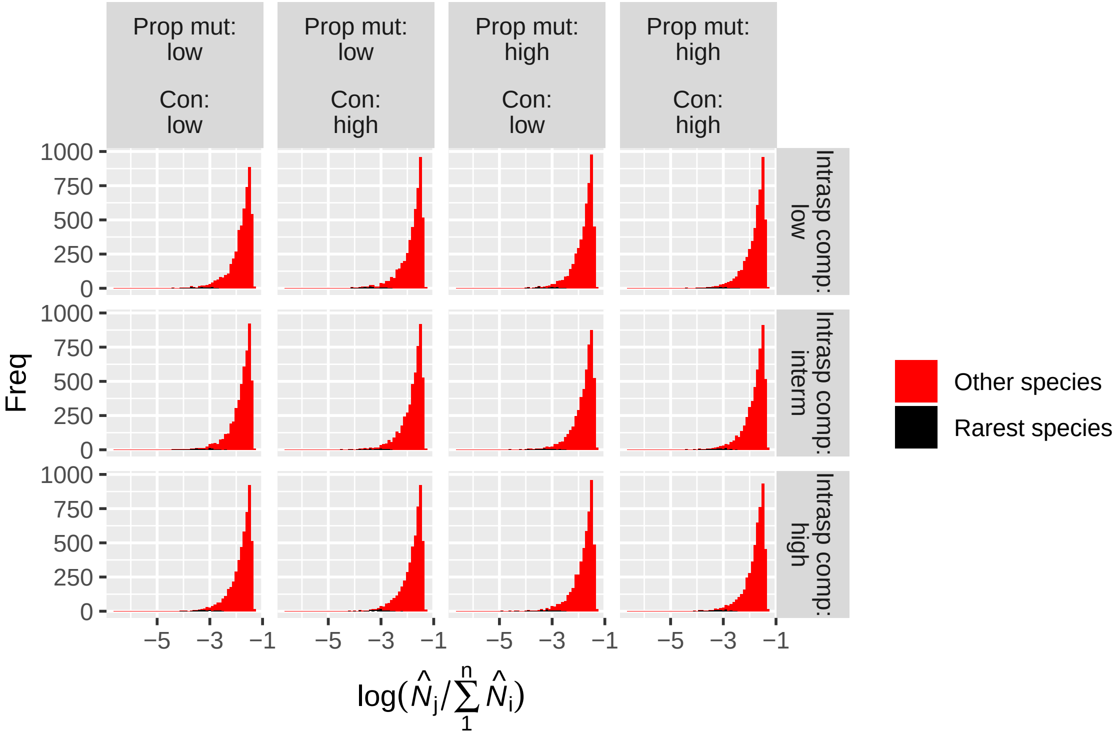
Prop mut: low
Con: low
Prop mut: low
Con: high
Prop mut: high
Con: low
Prop mut: high
Con: high
Intrasp comp: low
Intrasp comp: interm
Intrasp comp: high
Freq
1000
750
500
250
0
−5
−3
−1
$\log(\hat{N}_j / \sum_1^n \hat{N}_i)$
Other species
Rarest species

Supplementary Figure 13 | Equilibrium density distribution in bipartite networks. This histogram shows the relative distribution of equilibrium biomasses within bipartite network replicates ($\log\left(\frac{\hat{N}_j}{\sum_i \hat{N}_i}\right)$), for each network configuration. The rarest species in each food web replicate is coded in black and other species are coded in red. Three parameters are varied among subplots: Connectance – the proportion of possible links that are realised (low=0.3 and high=0.7); Proportion of mutualistic links – the proportion of all links where interacting species have positive effects on each other (low=0.3 and high=0.7); Intraspecific competition – a scaling parameter, ω, determining the average strength of species self-limitation (ω_{low}=0.5, $\omega_{\text{intermediate}}$=1 and ω_{high}=2).

Supplementary Tables

Supplementary Table 1 | Characteristics and references to food webs. Number of species *S* refers to the number of trophic species in each food web, connectance is the realized number of interactions out of all possible pair-wise consumer-resource interactions in a community, Ref refers to the references from which food web data was assembled.

Web	Number of species, S	Connectance	Ref. topology	Ref. body masses
Baltic Sea	9	0.19	17	17
Broadstone Stream	28	0.20	38	41
Lake Vättern	11	0.18	17	17
Montane Forest	28	0.06	39	42
Skipwith Pond	33	0.32	40	41
Trelease Woods	24	0.09	39	42
Tropic Sea	19	0.14	39	42

Supplementary Table 2 | Difference in equilibrium density between the most common and the rarest species in the bipartite networks. This table shows the mean and standard deviation of the differences in log equilibrium biomass between the most common and the rarest species in the bipartite networks; that is, $\log\left(\max_j \hat{N}_j\right) - \log\left(\min_j \hat{N}_j\right)$.

Intraspecific competition	Proportion mutualistic links	Connectance	mean	sd
0.5	0.3	0.3	2.11	0.63
0.5	0.3	0.7	2.09	0.54
0.5	0.7	0.3	1.92	0.51
0.5	0.7	0.7	2.01	0.61
1	0.3	0.3	2.05	0.53
1	0.3	0.7	1.96	0.53
1	0.7	0.3	1.96	0.52
1	0.7	0.7	1.91	0.49
2	0.3	0.3	1.89	0.5
2	0.3	0.7	1.86	0.46
2	0.7	0.3	2	0.64
2	0.7	0.7	1.92	0.52

1. The relationship between resilience and species densities

Two-species systems

We start by looking at the resilience of simple two-species communities:

$$\begin{cases} \dfrac{dN_1}{dt} = N_1\left(r_1 + \alpha_{11}N_1 + \alpha_{12}N_2\right) \\ \dfrac{dN_2}{dt} = N_2\left(r_2 + \alpha_{21}N_1 + \alpha_{22}N_2\right) \end{cases} \tag{A1}$$

Here r_1 is the intrinsic growth rate of species 1, r_2 is the intrinsic growth rate of species 2 and the α_{ij}*'s* are the per capita interaction strengths between and within species. The sign and magnitude of the parameters depends on the type of system; competitive (r_1>0 and r_2>0, α_{12}<0 and α_{21}<0), facultative mutualistic (r_1>0 and r_2>0, α_{12}>0 and α_{21}>0), resource-consumer or host-parasite (if species 1 is the prey or host; r_1>0 and r_2<0, α_{12}<0 and α_{21}>0). Per capita interaction strengths within species are negative (α_{11}<0 and α_{22}<0). The equilibrium densities of the species are; $\hat{N}_1 = \left(r_2\alpha_{12} - r_1\alpha_{22}\right)/D$ and $\hat{N}_2 = \left(r_1\alpha_{21} - r_2\alpha_{11}\right)/D$ where $D = \alpha_{11}\alpha_{22} - \alpha_{12}\alpha_{21}$ is the determinant of the interaction matrix, **A** (with elements α_{ij}). The local stability of this equilibrium is determined by the dominant eigenvalue (λ) of the Jacobian matrix, **C** (with elements $c_{ij} = \alpha_{ij}\hat{N}_i$), which is given by:

$$\lambda = \frac{B}{2}\left(1 - \sqrt{1 - \frac{4D}{B^2}\hat{N}_1\hat{N}_2}\right) \tag{A2}$$

Here $B = \alpha_{11}\hat{N}_1 + \alpha_{22}\hat{N}_2 < 0$. When $B^2 >> 4D\hat{N}_1\hat{N}_2$, λ will be real and close to zero (i.e., the system is close to instability). Resilience, $-\lambda$, is then approximately

$$\Lambda \approx -D\hat{N}_1\hat{N}_2 / B = -D\left(\alpha_{22}/\hat{N}_1 + \alpha_{11}/\hat{N}_2\right)^{-1} \tag{A3}$$

showing that the resilience of a system close to instability is determined by a weighted harmonic mean of the species equilibrium densities. This means that resilience tends to be predominantly determined by the species with the lowest density. For example, if species 1 is very rare compared to species 2 then

$$\Lambda \approx -\frac{D}{\alpha_{22}}\hat{N}_1, \qquad \text{(A4)}$$

approximating resilience as a linear function of the density of the rarest species.

Complex multi-species communities

The results for the simple two-species systems can be generalised to show that rare species can also govern the resilience of complex multi-species communities containing a variety of interaction types. This can be seen from the characteristic equation for an *n*-species community and its Taylor expansion in powers of λ using the Jacobi formula for the determinant of a matrix [ref S1]:

$$\det(\mathbf{C} - \lambda\mathbf{1}) = \sum_{\boldsymbol{x}=\mathbf{0}}^{\boldsymbol{n}} \frac{\varphi_{-x}}{x!}(-\lambda)^x = 0 \qquad \text{(A5)}$$

Here φ_{-x} is a sum of determinants, where the determinants are all possible combinations of the Jacobian matrix **C** with *x* pairs of rows–columns removed. Equation (A5) can be rewritten in terms of the interaction matrix **A** (with elements α_{ij}) as

$$D - \lambda\sum_{i=1}^{n}\frac{D_{-i}}{\hat{N}_i} + \frac{\lambda^2}{2}\sum_{\substack{i,j=1\\ j\neq i}}^{n}\frac{D_{-ij}}{\hat{N}_i\hat{N}_j} + O(\lambda^3) = 0 \qquad \text{(A6)}$$

Here D is the determinant of the Lotka-Volterra interaction matrix **A** with elements α_{ij} (eq. A1), D_{-i} is the determinant of the reduced matrix **A**-i with the *i*th row and column removed from **A**, and D_{-ij} is the determinant of the reduced matrix **A**-ij with the *i*th and *j*th rows and columns removed from

A. When λ is sufficiently close to zero (i.e., the system is close to instability) higher order terms can be ignored, giving

$$\lambda \approx \frac{\Delta_{-1}}{\Delta_{-2}}\left(1-\sqrt{1-\frac{2D\Delta_{-2}}{\Delta_{-1}{}^{2}}}\right) \qquad \text{(A7)}$$

Here $\Delta_{-1}=\sum_i D_{-i}/\hat{N}_i$ and $\Delta_{-2}=\sum_{ij,j\neq i} D_{-ij}/\left(\hat{N}_i\hat{N}_j\right)$. When $2|D||\Delta_{-2}| << \Delta_{-1}{}^{2}$ the square root in equation (A7) is positive and the network resilience can be approximated by

$$\Lambda \approx -D/\Delta_{-1} = -D\left(\sum_i D_{-i}/\hat{N}_i\right)^{-1} \qquad \text{(A8)}$$

This is the generalisation of equation (A3) for large systems. It shows that resilience is a weighted harmonic mean of the species abundances, and therefore tends to be dominated by the rare species. Now, if species 1 is the rarest species and further $\hat{N}_1\big/\hat{N}_j << |D_{-1}|/|D_{-j}|$ and $\hat{N}_1\big/\hat{N}_j << D_{-1}{}^{2}/|DD_{-1j}|$ for all j>1, then resilience can be further approximated by

$$\Lambda \approx -\frac{D}{D_{-1}}\hat{N}_1 \qquad \text{(A9)}$$

This approximation shows that resilience is most sensitive to changes in the equilibrium density of the rarest species in a community.

When $2D\Delta_{-2} > \Delta_{-1}{}^{2}$ the square root in equation (A7) is imaginary, network resilience is given by $\Lambda \approx -\frac{\Delta_{-1}}{\Delta_{-2}}$. Letting species 1 be the rarest species and species 2 be the next rarest species, the resilience becomes $\Lambda \approx -D_{-1}\left[\sum_{j\neq 1} D_{-1j}/\hat{N}_j\right]^{-1} \approx -D_{-1}\hat{N}_2/D_{-12}$, showing that the stability of the system is still a harmonic mean of species abundances but now excluding species 1, and that

resilience is still primarily determined by rare species (in this case species 2, the second rarest species).

2. Solution to systems of linear differential equations

The linear dynamical system:

$$\frac{d\mathbf{x}}{dt} = \mathbf{C}\mathbf{x}, \qquad \text{(B1)}$$

where **C** is a matrix with coefficients, and **x** is a vector of state variables, has the particular solution:

$$\mathbf{x}(t) = e^{\mathbf{C}t}\mathbf{x}(0), \qquad \text{(B2)}$$

where **x**(0) are the initial conditions at time *t*=0. Following a diagonalization of matrix **C**, equation (B2) can be rewritten as:

$$\mathbf{x}(t) = \mathbf{V}e^{\mathbf{\Lambda t}}\mathbf{V}^{-1}\mathbf{x}(0), \qquad \text{(B3)}$$

where **Λ** is a diagonal matrix with eigenvalues, λ_i, along its diagonal; **V** is a matrix with right eigenvectors $\vec{\mathbf{v}_i}$ as columns; and $\mathbf{V}^{-1}$ a matrix with rows being the left eigenvectors, $\vec{\mathbf{w}_i}$, of matrix **C**.

As an example, consider a system consisting of two interacting species. Its solution is given by:

$$\mathbf{x}(\mathrm{t}) = \begin{bmatrix} x_1(0)\left(\mathbf{v}_{1(1)}e^{\lambda_1 t}\mathbf{w}_{1(1)} + \mathbf{v}_{2(1)}e^{\lambda_2 t}\mathbf{w}_{2(1)}\right) + x_2(0)\left(\mathbf{v}_{1(1)}e^{\lambda_1 t}\mathbf{w}_{1(2)} + \mathbf{v}_{2(1)}e^{\lambda_2 t}\mathbf{w}_{2(2)}\right) \\ x_1(0)\left(\mathbf{v}_{1(2)}e^{\lambda_1 t}\mathbf{w}_{1(1)} + \mathbf{v}_{2(2)}e^{\lambda_2 t}\mathbf{w}_{2(1)}\right) + x_2(0)\left(\mathbf{v}_{1(2)}e^{\lambda_1 t}\mathbf{w}_{1(2)} + \mathbf{v}_{2(2)}e^{\lambda_2 t}\mathbf{w}_{2(2)}\right) \end{bmatrix}, \qquad \text{(B4)}$$

where $\mathbf{V} = [\overrightarrow{\mathbf{v}_1},\overrightarrow{\mathbf{v}_2}]$; $e^{\mathbf{\Lambda t}} = \begin{bmatrix} e^{\lambda_1 t} & 0 \\ 0 & e^{\lambda_2 t} \end{bmatrix}$; $\mathbf{V}^{-1} = \begin{bmatrix} \overrightarrow{\mathbf{w}_1} \\ \overrightarrow{\mathbf{w}_2} \end{bmatrix}$; $\mathbf{x}(0) = \begin{bmatrix} x_1(0) \\ x_2(0) \end{bmatrix}$; and $\mathbf{w}_{i(j)}$ is the *j*th element of the *i*th left eigenvector.

In matrix notation the solution of the two dimensional system (B4) can be described as follows:

$$\mathbf{x}(\mathrm{t}) = k_1\overrightarrow{\mathbf{v}_1}e^{\lambda_1 t} + k_2\overrightarrow{\mathbf{v}_2}e^{\lambda_2 t}, \qquad \text{(B5)}$$

where k_1 is a linear combination of initial conditions, i.e. $\mathbf{x}(0)$, and the first left eigenvector, i.e. $\overrightarrow{\mathbf{w}_1}$; k_2 is a linear combination of initial conditions, i.e. $\mathbf{x}(0)$, and the second left eigenvector, i.e. $\overrightarrow{\mathbf{w}_2}$; $\overrightarrow{\mathbf{v}_i}$ is the *i*th right eigenvector; and λ_i is the *i*th eigenvalue.

If λ_1 is the dominant eigenvalue, i.e. the eigenvalue with the largest real part, this equation shows that initially, just after a perturbation has been imposed [i.e. at $t=0^+$], species densities are dependent on all eigenvalues. Yet, as *t* increases, the solution of the system, i.e. species densities, will be dominated by the dominant eigenvalue.

Equation (B5) further illustrates that for the part of species densities determined by one eigenvalue λ_i, its associated right eigenvector $\overrightarrow{\mathbf{v}_i}$ determines how species densities distribute, and the left eigenvector $\overrightarrow{\mathbf{w}_i}$ in combination with initial conditions $\mathbf{x}(0)$ determines how strongly a perturbation initially affects all species in a community.

3. Natural food webs

Natural food webs were chosen to represent a number of different ecosystem types, ranging from freshwater, marine, and terrestrial habitats. The original references including body masses and food web topology information are shown in Supplementary Table 1.

4. Bipartite model networks with a mixture of mutualistic and antagonistic interactions

The dynamics of bipartite model networks is represented by continuous time models with type 1 functional response[30]:

$$dN_i/dt = N_i(r_i - \alpha_{ii}N_i + \sum_{j,j\neq i} \tilde{\alpha}_{ij}\, N_j) \qquad \text{(C1)}$$

Here N_i is the density of species i, r_i is the intrinsic growth rate of species i, α_{ii} is the strength of intraspecific competition and $\tilde{\alpha}_{ij}$ is the interaction strength between species i and j. For functional response of type 1 the interspecific interaction strengths, $\tilde{\alpha}_{ij}$, are given by:

$$\begin{cases} \tilde{\alpha}_{ij} = f^{(\mathrm{B})}P_{ij}^{(\mathrm{B})} & \tilde{\alpha}_{ij} \in \mathrm{B} \\ \tilde{\alpha}_{ij} = -f^{(\mathrm{A})}P_{ij}^{(\mathrm{A})} & \tilde{\alpha}_{ij} \in \mathrm{A} \\ \tilde{\alpha}_{ji} = e_{ij}f^{(\mathrm{A})}P_{ij}^{(\mathrm{A})} & \tilde{\alpha}_{ji} \in \mathrm{A} \\ \tilde{\alpha}_{ij} = g_{ij}f^{(\mathrm{M})}P_{ij}^{(\mathrm{M})} & \tilde{\alpha}_{ij} \in \mathrm{M} \\ \tilde{\alpha}_{ji} = g_{ji}f^{(\mathrm{M})}P_{ji}^{(\mathrm{M})} & \tilde{\alpha}_{ji} \in \mathrm{M} \end{cases} \qquad \text{(C2)}$$

Here, B is the set of competitive interactions among basal species, A is the set of antagonistic interactions, M is the set of mutualistic interactions, e_{ij} and g_{ij}, are conversion efficiencies for antagonistic and mutualistic interactions, respectively and $f^{(B)}$, $f^{(A)}$ and $f^{(M)}$ are the relative strengths of competitive, antagonistic and mutualistic interactions, respectively. The preferences (allocation of interaction efforts) P_{ij} are given by:

$$P_{ij} = I_{ij}/\sum_k I_{kj} \text{ where } I_{ij} \in [0,1]. \qquad \text{(C3)}$$

Here I_{ij} is the potential preference of species j for the interaction partner i, k is the resource species of species j in the sets of mutualistic or antagonistic interactions, respectively. For basal species k is the set of competitors of species j. For each species j these preferences sum up to 1. Thus, the effect of species j on species i will be weaker, on average, if species j interacts with many other species than if it interacts with few other species (See [ref. no 30] for details). Parameters e_{ij} and g_{ij} are drawn from the uniform interval [0, 1], $f^{(B)}$, $f^{(A)}$ and $f^{(M)}$

are all set to 1 and α_{ii} is drawn from the uniform interval ω[0, 1], where ω (set to 0.5, 1 or 2) determines the relative strength of self-regulation.

Equilibrium densities of species ($\widehat{N}_i$) are drawn from the uniform interval [0, 1] and intrinsic rates of change are solved for; $r_i = \alpha_{ii}\widehat{N}_i - \sum_{j\neq i} \tilde{\alpha}_{ij}\widehat{N}_j$. Local stability of the communities is assessed by checking that all eigenvalues of the Jacobian matrix have negative real parts. For each combination of proportion of mutualistic interactions (0.3 and 0.7), connectance (0.3 and 0.7) and strength of intraspecific competition, ω (0.5, 1 and 2), we assembled 100 locally stable network replicates, each containing 50 species.

5. Initial return rate following selective pulse perturbations

Consider the dynamics of a linearized dynamical system:

$$\frac{d\mathbf{x}}{dt} = \mathbf{Cx}, \qquad \text{(D1)}$$

where **C** is a Jacobian matrix evaluated at a locally stable equilibrium, and **x** is a vector of displacements ($x_i = N_i - \widehat{N}_i$) from a locally stable state $\widehat{\mathbf{N}}$.

The growth rate of a small perturbation to the locally stable equilibrium can be described by (See ref S2):

$$\frac{d\|\mathbf{x}\|}{dt} = \frac{d\sqrt{\mathbf{x}^{\mathrm{T}}\mathbf{x}}}{dt} = \frac{\mathbf{x}^{\mathrm{T}}\left(\frac{d\mathbf{x}}{dt}\right)+\left(\frac{d\mathbf{x}}{dt}\right)^{\mathrm{T}}\mathbf{x}}{2\|\mathbf{x}\|} = \frac{\mathbf{x}^{\mathrm{T}}(\mathbf{C}+\mathbf{C}^{T})\mathbf{x}}{2\|\mathbf{x}\|}, \qquad \text{(D2)}$$

where $(\mathbf{C} + \mathbf{C}^T)/2$ is called the Hermitian part of **C**, here annotated H(**C**).

At time *t*=0, the per capita growth rate of a perturbation is given by:

$$\left(\frac{1}{\|\mathbf{x}\|}\frac{d\|\mathbf{x}\|}{dt}\right)\Big|_{t=0} = \frac{\mathbf{x_0}^{\mathrm{T}}H(\mathbf{C})\mathbf{x_0}}{\mathbf{x_0}^{\mathrm{T}}\mathbf{x_0}}. \qquad \text{(D3)}$$

Now, if a selective perturbation of magnitude $||\mathbf{x}_o|| = 1$ is assumed (i.e. a perturbation affecting one species only), the initial perturbation vector, $\mathbf{x}_o$, is a zero vector with one in its *i*th element. A selective perturbation to species *i* therefore yields an initial recovery rate of the community following:

$$\left(\frac{1}{||\mathbf{x}||}\frac{d||\mathbf{x}||}{dt}\right)\Big|_{t=0} = -\frac{\mathbf{x_0}^{\mathrm{T}}H(\mathbf{C})\mathbf{x_0}}{\mathbf{x_0}^{\mathrm{T}}\mathbf{x_0}} = -\frac{(\mathrm{c}_{ii}+\mathrm{c}_{ii})}{2} = -\mathrm{c}_{ii} = -\alpha_{ii}\widehat{N}_i, \qquad \text{(D4)}$$

where c_{ii} is the i:th diagonal element of the Jacobian matrix, α_{ii} is the i:th diagonal element of the Lotka-Volterra interaction matrix and $\widehat{N}_i$ is the equilibrium density of species *i*. If it is assumed that intraspecific competition coefficients, α_{ii}, in a community are approximately equal, the initial rate at which a community recovers following a selective perturbation will be slower if the perturbation affects a rare rather than a common species.

6. Analytical result illustrating the relative effect of press perturbations to rare and common species

The Jacobian Matrix **C** of a generalized Lotka-Volterra system evaluated at an equilibrium point (with elements given by $c_{ij} = \frac{\partial \frac{dN_i}{dt}}{\partial N_j}\Big|_{\widehat{\mathbf{N}}}$) can be decomposed into two matrices:

$$\mathbf{C} = \mathrm{diag}(\widehat{\mathbf{N}}) * \mathbf{A}, \qquad \text{(E1)}$$

where **C** is the Jacobian Matrix, $\mathrm{diag}(\widehat{\mathbf{N}})$ is a diagonal matrix with species equilibrium densities ($\widehat{\mathbf{N}}$) along the diagonal, and **A** is the Lotka-Volterra interaction strength matrix (where elements α_{ij} represent the per-capita interaction strength between species *j* and species *i*).

Further, the negative inverse of the Jacobian matrix (See Supporting Information Section 9) is given by:

$$-\mathbf{C}^{-1} = -(\mathrm{diag}(\widehat{\mathbf{N}}) * \mathbf{A})^{-1} = -\mathbf{A}^{-1}(\mathrm{diag}(\widehat{\mathbf{N}}))^{-1}. \qquad \text{(E2)}$$

The elements along the diagonal of an inverse diagonal matrix (which itself is a diagonal matrix) are given by $(\mathrm{diag}(\widehat{\mathbf{N}})^{-1})_{ii} = 1/\widehat{N}_{ii}$, thus giving:

$$(-\mathbf{C}^{-1})_{ij} = -\frac{\gamma_{ij}}{\widehat{N}_j}, \qquad \text{(E3)}$$

where γ_{ij} is the *i-j*th element of the inverse interaction matrix $\mathbf{A}^{-1}$ and $\widehat{N}_j$ is the equilibrium density of species *j*.

Since the effect of a press perturbation of a given species *j* on another species *i* is given by the *i-j*th element of the inverse Jacobian matrix, the effect of a given press perturbation is related to the equilibrium density of the perturbed species (eq. E3). Thus, if there is no specific patterning in the inverse interaction strength matrix $\mathbf{A}^{-1}$, equation (E3) shows that selective press perturbations to rare species in general lead to larger effects within a system than perturbations to common species.

7. Localization of left and right eigenvectors when one species is rare

The eigenvectors associated with different eigenvalues give information about how pulse perturbations hit and propagate within a community (ref S3; See also Supplementary Information Section 2). Information encoded in the eigenvectors thus tells us how strongly a system is initially hit by a pulse perturbation (left eigenvectors), and to what extent a pulse perturbation will propagate (right eigenvectors) in a system before it eventually decays. If the left eigenvector is localized, i.e. concentrated on specific elements in the eigenvector, perturbations affecting that specific species will have a more pronounced effect on all species densities than if the perturbation hit another species in the system (See eq. B4 in

Supplementary Information Section 2). By contrast, the right eigenvectors encodes how a perturbation will distribute proportionally as time goes to infinity (i.e. t -> ∞) (See eq. B4 in Supplementary Information Section 2; ref S3). Below we show that for a system close to an instability; that is, a system in which rare species primarily govern resilience, the dominating left eigenvector is concentrated on the rarest species in the system, and that there is no reason to believe that the dominating right eigenvector should be localized. This infers that pulse perturbations affecting rare species will hit the system to a larger extent than pulse perturbation affecting common species. Further, this infers that pulse perturbations will propagate among all species in the system since the right eigenvector is non-localized. Overall, these results infer that rare species are particularly important for the propagation of perturbations since selective perturbations affecting rare species will have a stronger and more system wide effect than perturbations affecting common species.

Left eigenvector

In section 1 of the Supplement Information we showed that the dominating eigenvalue (of a real eigenvalue) of a locally stable system that is close to an instability can be approximated by:

$$\lambda \approx \frac{D}{D_{-1}} \widehat{N}_1 \qquad \text{(F1)}$$

Where D is the determinant of the interaction matrix **A**, and D_{-1} is the determinant of the reduced interaction matrix $\mathbf{A}_{-i}$ with column and row i removed. To simplify the expressions we write $k_1 = \frac{D}{D_{-1}}$ (Note that k_1 is positive since we are assuming locally stable communities).

The dominant left eigenvector is found by solving the following set of equations:

$$\sum_{i=1}^{n} w_i \alpha_{ij} \hat{N}_i = \lambda w_j \; for \; (w_j, j = 1, \dots, n) \quad \text{(F2)}$$

Where w_i is the ith element of the dominant left eigenvector, α_{ij} is the i-jth element of the interaction matrix **A** and $\hat{N}_i$ is the equilibrium density of species i.

Substituting in equation (F1) and rewriting equation (F2) yields:

$$\sum_{i=1}^{n} \hat{N}_i (\alpha_{ij} + k_1 \frac{\hat{N}_1}{\hat{N}_i} \delta_{ij}) w_i = 0 \quad \text{(F3)}$$

Where $\delta_{ij} = 1$ when i=j, and 0 otherwise. After writing $\hat{N}_i w_i = W_i$, then in matrix form equation (F3) can be written as:

$$\mathbf{WM} = \mathbf{0}, \quad \text{(F4)}$$

where $M_{ij} = \alpha_{ij} + k_1 \frac{\hat{N}_1}{\hat{N}_i} \delta_{ij}$. If one species is rare compared to other species (i.e. $\hat{N}_1 \ll \hat{N}_i$), then elements of **M** have little direct dependence on equilibrium abundance, and therefore the elements of the left eigenvector, w_i, are almost inversely proportional to $\hat{N}_i$. Thus, if one species is rare the dominant left eigenvector is concentrated on this species.

Right eigenvector

If it is assumed that the dominating eigenvalue is primarily determined by the abundance of the rarest species in a community (eq. F1), the right eigenvector can be found by solving:

$$\sum_{j=1}^{n} \alpha_{ij} \hat{N}_i v_j = \lambda v_i, \quad \text{(F5)}$$

where v_i is the ith element of the right eigenvector.

Rewriting and inserting equation (F1) in equation (F5) gives:

$$\sum_{j=1}^{n} \hat{N}_i \left(\alpha_{ij} + k_1 \frac{N_1^*}{N_i^*} \delta_{ij} \right) v_j = 0, \quad \text{(F6)}$$

which in matrix form can be written as

$$\hat{\mathbf{N}}' \mathbf{M} \mathbf{v} = 0 \quad \text{(F7)}$$

Since $\hat{N}_i \neq 0$, equation (F7) has a solution when

$$\mathbf{M} \mathbf{v} = 0 \quad \text{(F8)}$$

As matrix **M** has little direct dependence on species´ equilibrium abundances, there is no specific reason to believe that the dominant right eigenvector **v** is localized, i.e. that some elements are much larger than other elements.

8. Sensitivity of resilience

Consider a network whose dynamics is described by a generalized Lotka-Volterra model:

$$dN_i/dt = N_i(r_i + \sum_j \alpha_{ij} N_j) \quad \text{(G1)}$$

Here dN_i/dt is the rate of change in density of species *i*, N_i is the density of species *i*, r_i is the intrinsic growth rate of species *i*, α_{ij} is the per capita effect of species *j* on the per capita growth rate of species *i*. The equilibrium density of species *i* is given by: $\hat{N}_i = -\sum_j r_j \gamma_{ij}$, where γ_{ij} is element *ij* of the inverse interaction matrix of the network[35].

The resilience (the asymptotic rate of return to equilibrium following a temporary perturbation) of the network is a function of the dominant eigenvalue of the Jacobian matrix (with elements $c_{ij} = \alpha_{ij} \hat{N}_i$). Now, the sensitivity of the dominant eigenvalue, λ, of the Jacobian matrix to a small absolute perturbation to the equilibrium density of species *k*, $\hat{N}_k$, is given by:

$$S_\lambda(\hat{N}_k) = \partial\lambda/\partial\hat{N}_k = \sum_{i,j} \frac{\partial\lambda}{\partial c_{ij}} \frac{\partial c_{ij}}{\partial\hat{N}_k} \quad \text{(G2)}$$

Next, since $c_{ij} = \alpha_{ij}\hat{N}_i$, we have:

$$S_\lambda(\hat{N}_k) = \sum_{i,j} \frac{\partial\lambda}{\partial c_{ij}} \alpha_{ij} \frac{\partial\hat{N}_i}{\partial\hat{N}_k} \quad \text{(G3)}$$

Here $\frac{\partial\lambda}{\partial c_{ij}} = \frac{\bar{v}_i w_j}{\langle \mathbf{w},\mathbf{v}\rangle}$ where **v** and **w** are the left and right eigenvectors corresponding to the dominant eigenvalue of the Jacobian matrix, v_i and w_j are elements i and j of the respective vectors, $\bar{v}_i$ is the complex conjugate of v_i, and < **w, v** > is the scalar product of **w** and **v**.

Now, since $\partial\hat{N}_i/\partial\hat{N}_k = 0$ for all $i \neq k$ and equal to 1 when $i = k$ we have that

$$S_\lambda(\hat{N}_k) = \sum_j \frac{\bar{v}_k w_j}{\langle \mathbf{w},\mathbf{v}\rangle} \alpha_{kj} \quad \text{(G4)}$$

Since resilience (Λ) is the negative of the real part of the dominant eigenvalue, i.e. Λ = -Re(λ), the sensitivity of resilience with respect to a change in the equilibrium density of species k, $\hat{N}_k$, is given by:

$$S_\Lambda(\hat{N}_k) = -\mathrm{Re}\left(\sum_j \frac{\bar{v}_k w_j}{\langle \mathbf{w},\mathbf{v}\rangle} \alpha_{kj}\right) \quad \text{(G5)}$$

Here we assume that it is the intrinsic growth rates, i.e. r_i, that change inducing a small permanent change in the equilibrium density of species k.

Finally, the elasticity of resilience, Λ, to a small proportional perturbation to the equilibrium density of species k, $\hat{N}_k$, is given by:

$$E_\Lambda(\hat{N}_k) = -\mathrm{Re}\left(\sum_j \frac{\bar{v}_k w_j}{\langle \mathbf{w},\mathbf{v}\rangle} \alpha_{kj}\right)\left(\frac{\hat{N}_k}{\Lambda}\right) \quad \text{(G6)}$$

9. Interpretation of the inverse Jacobian matrix

Mathematically, the inverse Jacobian matrix can be derived by perturbing a linearized system by a small continuous density addition to one species (population density/unit time) in a community. This is given by:

$$\frac{d\mathbf{x}}{dt} = \mathbf{Cx} + \mathbf{e}, \qquad \text{(H1)}$$

where **C** is the Jacobian matrix evaluated at an equilibrium point, **x** is species displacements from the equilibrium, i.e. $x_i = N_i - \widehat{N}_i$, and **e** is a zero vector with a small positive value ε_j in its *j*th element. Now, solving for the equilibrium of equation (H1) yields:

$$\hat{\mathbf{x}} = -\mathbf{C}^{-1}\mathbf{e} \qquad \text{(H2)}$$

Further, differentiating equation (H2) with respect to ε_j gives:

$$\frac{\partial \hat{\mathbf{x}}}{\partial \varepsilon_j} = \begin{bmatrix} -(c_{1j})^{-1} \\ \vdots \\ -(c_{nj})^{-1} \end{bmatrix}, \qquad \text{(H3)}$$

where $(c_{ij})^{-1}$ is the *i-j*th element of the inverse Jacobian matrix $\mathbf{C}^{-1}$. Equation (H3) shows that the elements (*i*,*j*) of the negative inverse Jacobian matrix translates into the sensitivity of species *i*'s equilibrium density to a small continuous density addition of species *j*.

10. Supplementary references

S1. Jeffrey, A. & Dai, H. H. *Handbook of Mathematical Formulas and Integrals* (Academic Press, Amsterdam, 2008).

S2. Neubert, M. & Caswell, H. Alternatives to resilience for measuring the responses of ecological systems to perturbations. *Ecology* **78**, 653-665 (1997).

S3. Suweis, *et al.* Effect of localization on the stability of mutualistic ecological networks. *Nat. Commun.* **6**, 10179 (2015).
